# Supplementary material for: Barriers for tuberculosis case finding in Southwest Ethiopia: A qualitative study
Source: PLoS One. 2020 Jan 2;15(1):e0226307. doi: 10.1371/journal.pone.0226307 (PMC6939902; doi:10.1371/journal.pone.0226307)
Supplement: S1 Text — (PDF) [file pone.0226307.s001.pdf]

Information sheets and consent forms

1. Request for participation in a research project for local and regional health office program managers/coordinators

"Performance and Quality of Tuberculosis Directly Observed Treatment Short Course (DOTS) Strategy in Jimma Zone, Southwest Ethiopia"

Introduction

My name is ____________________________ I am PhD student at the University of Oslo, Norway. I am kindly inviting you, to participate in a research study because you have been implementing tuberculosis prevention and control program and have experience in the area. The details of the research plan are described in this document. It is important that you understand why the research is being done and what it will involve. Please take your time to read through and consider this information carefully before you decide to participate in the proposed study. Please, ask if anything is unclear or if you would like to get more information.

Background and purpose

Tuberculosis (TB) is an infectious disease, responsible for serious illness and death globally, affecting both sexes of all age groups. TB is a common problem in Ethiopia including Jimma Zone. It is important to control the disease with feasible, cost effective, and acceptable approaches. The research project intends to assess the overall performance and quality of tuberculosis directly observed treatment short course (TB DOTS) strategy in Jimma Zone, Ethiopia. The knowledge obtained from this study will provide relevant information that may help you to improve the performance and quality of the program. Due to your position as a health office program manager/coordinator you have participated in many of the overall tuberculosis program related activities and we believe that you have ample knowledge in regards to how the program is implemented. You are therefore selected purposefully as we consider that you can provide relevant knowledge for our study.

What does the study entail?

In this study I would like to interview you about your experience and views of tuberculosis directly observed treatment short course. The interview will last about an hour and take place at a time/place convenient for you. If you are willing to participate, the interview will be tape recorded. I would like to ask you a few questions about the TB control program to explore potential barriers and facilitators during the implementation of this program. Whether you participate or not will not in any way affect your position.

Potential advantages and disadvantages

You may spend about an hour providing us information regarding to TB DOTS strategy which may consume your precious time. There may not be any direct benefit as a result of your participation in the study, however your honest and genuine response will contribute to generate

information that can be used to improve performance and quality of services related to tuberculosis.

What will happen to information about you?

The data that are registered about you will only be used in accordance with the purpose of the study. All the data will be processed without name, ID number or other directly recognisable type of information. A code number that links you to your data will be used, and the information you provide us will therefore be confidential. All data will be kept in a locked cabinet and pass word protected computers. In addition, your information will only be used during report writing and not after completing the project.

Voluntary participation

Participation in the study is voluntary. You can withdraw your consent to participate in the study at any time and without stating any particular reason. This will not have any consequences for your further position. If you wish to participate, sign the declaration of consent on the final page.

If you agree to participate at this time, you may later on withdraw your consent without your position being affected in any way. If you later on wish to withdraw your consent or have questions concerning the study, you may contact:

Berhane Megerssa Ereso
Mobile phone number +251917804469
Email address – berhanemegerssa2004@gmail.com
Jimma University, Ethiopia

Supervisors

1. Mette Sagbakken (PhD, Associate professor)
Email address mette.sagbakken@nakmi.no
Mobile phone number +4741576964
2. Solomon Yimer (PhD, Postdoc)
Email address yimsolo@yahoo.com
Mobile phone number +4747687670

Releasing material and data to other parties

If you agree to participate in the study, you also consent de-identified data being released to local and regional health offices in Ethiopia.

Right to access and right to delete your data

If you agree to participate in the study, you are entitled to have access to what information is registered about you. You are further entitled to correct any mistakes in the information we have registered. If you withdraw from the study, you are entitled to demand that the collected data are deleted, unless the data have already been incorporated in analyses or used in scientific publications.

Funding and the role of Strategic and Collaborative Capacity Development in Ethiopia and Africa (SACCADE) Project

The study will be supported by the Strategic and Collaborative Capacity Development in an Ethiopian and African (SACCADE) project, University of Oslo. The SACCADE project will cover expenses related with personal, materials/ supplies and transportation. It does not have any conflict of interest with any other project.

Information about the outcome of the study

You are fully entitled to receive information about the result and outcome of this study.

Consent for participation in the study

I am willing to participate in the study

(Signature of the study participant, date)

I confirm that I have given information about the study.

(Signature of the data collector, date)

2. Request for participation in a research project for tuberculosis directly observed treatment short course (DOTS) providers

"Performance and Quality of Tuberculosis Directly Observed Treatment Short Course (DOTS) Strategy in Jimma Zone, Southwest Ethiopia*"*

Introduction

My name is ____________________________I am PhD student at the University of Oslo, Norway. I am kindly inviting you, to participate in a research study because you have been implementing tuberculosis prevention and control program and have experience in the area. The details of the research plan are described in this document. It is important that you understand why the research is being done and what it will involve. Please take your time to read through and consider this information carefully before you decide to participate in the proposed study. Please, ask if anything is unclear or if you would like to get more information.

Background and purpose

Tuberculosis (TB) is an infectious disease, responsible for serious illness and death globally, affecting both sexes of all age groups. TB is a common problem in Ethiopia including Jimma Zone. It is important to control the disease with feasible, cost effective, and acceptable approaches. The research project intends to assess the overall performance and quality of tuberculosis directly observed treatment short course (TB DOTS) strategy in Jimma Zone,

Ethiopia. The knowledge obtained from this study will provide relevant information that will help you to improve the performance and quality of the program. Due to your position as a DOTS provider, you have participated in many of the overall tuberculosis program related activities and we believe that you have ample knowledge in regards to how the program is implemented. You are therefore selected purposefully as we consider that you can provide relevant knowledge for our study

What does the study entail?

In this study I would like to interview you about your experience and views of tuberculosis directly observed treatment short course. The interview will last about an hour and take place at a time/place convenient for you. If you are willing to participate, the interview will be tape recorded. I would like to ask you a few questions about the TB control program to explore potential barriers and facilitators during the implementation of this program. Whether you participate or not will not in any way affect your position.

Potential advantages and disadvantages

You may spend about an hour providing us information regarding to TB DOTS strategy which may consume your precious time. There may not be any direct benefit as a result of your participation in the study, however your honest and genuine response will contribute to generate information that can be used to improve performance and quality of services related to tuberculosis.

What will happen to information about you?

The data that are registered about you will only be used in accordance with the purpose of the study. All the data will be processed without name, ID number or other directly recognisable type of information. A code number that links you to your data will be used, and the information you provide us will therefore be confidential. All data will be kept in a locked cabinet and password protected computers. In addition, your information will only be used during report writing and not after completing the project.

Voluntary participation

Participation in the study is voluntary. You can withdraw your consent to participate in the study at any time and without stating any particular reason. This will not have any consequences for your further career. If you wish to participate, sign the declaration of consent on the final page. If you agree to participate at this time, you may later on withdraw your consent without any consequence. If you later on wish to withdraw your consent or have questions concerning the study, you may contact:

Berhane Megerssa Ereso
Mobile phone number +251917804469
Email address – berhanemegerssa2004@gmail.com
Jimma University, Ethiopia

Supervisors

1. Mette Sagbakken (PhD, Associate professor)
Email address mette.sagbakken@nakmi.no
Mobile phone number +4741576964
2. Solomon Yimer (PhD, Postdoc)
Email address yimsolo@yahoo.com
Mobile phone number +4747687670

Releasing material and data to other parties

If you agree to participate in the study, you also consent de-identified data being released to local and regional health offices in Ethiopia.

Right to access and right to delete your data

If you agree to participate in the study, you are entitled to have access to what information is registered about you. You are further entitled to correct any mistakes in the information we have registered. If you withdraw from the study, you are entitled to demand that the collected data are deleted, unless the data have already been incorporated in analyses or used in scientific publications.

Funding and the role of Strategic and Collaborative Capacity Development in Ethiopia and Africa (SACCADE) Project

The study will be supported by the Strategic and Collaborative Capacity Development in an Ethiopian and African (SACCADE) project, University of Oslo. The SACCADE project will cover expenses related with personal, materials/ supplies and transportation. It does not have any conflict of interest with any other project

Information about the outcome of the study

You are fully entitled to receive information about the result and outcome of this study.

Consent for participation in the study

I am willing to participate in the study

(Signature of the study participant, date)

I confirm that I have given information about the study.

(Signature of the data collector, date)

3. Request for participation in a research project for tuberculosis patients (for in-depth interview)

"Performance and Quality of Tuberculosis Directly Observed Treatment Short Course (DOTS) Strategy in Jimma Zone, Southwest Ethiopia"

Introduction

My name is ____________________________I am PhD student at the University of Oslo, Norway.

I am kindly inviting you, to participate in a research study because you have been diagnosed to have a disease called tuberculosis (TB). The details of the research plan are described in this document. It is important that you understand why the research is being done and what it will involve. Please take your time to read through and consider this information carefully before you decide to participate in the proposed study. Please, ask if anything is unclear or if you would like to get more information.

Background and purpose

Tuberculosis (TB) is an infectious disease, responsible for serious illness and death globally, affecting both sexes of all age groups. TB is a common problem in Ethiopia including Jimma Zone. It is important to control the disease with feasible, cost effective, and acceptable approaches. The research project intends to assess the overall performance and quality of tuberculosis directly observed treatment short course (TB DOTS) strategy in Jimma Zone, Ethiopia. The knowledge obtained from this study will help us to provide useful information for decision makers so that the program performance and quality can be improved and the community members get better services. To be able to do this, learning about your experience and your knowledge as being a TB patient under treatment is very important. You are therefore selected purposefully as we consider that you can provide relevant knowledge for our study

What does the study entail?

In this study I would like to interview you about tuberculosis directly observed treatment short course since you have been receiving the treatment. I would like to ask you a few questions about your experience and views of tuberculosis care to identify barriers and facilitators during receiving tuberculosis care. The interview will last about an hour and take place at a time/place convenient for you.If you are willing to participate, the interview will tape recorded. This study does not affect your treatment and you can receive your usual treatment whether you have participated in the study or not.

Potential advantages and disadvantages

You may spend about an hour providing us information about tuberculosis diagnosis & treatment which may consume your precious time and energy. There may not be any direct benefit as a result of your participation in the study, however your honest and genuine response will contribute to generate information that can be used to improve performance and quality of services related to tuberculosis.

What will happen to information about you?
The data that are registered about you will only be used in accordance with the purpose of the study. All the data will be processed without name, ID number or other directly recognisable type of information. A code number that links you to your data will be used, and the information you provide us will therefore be confidential. All data will be kept in a locked cabinet and password protected computers. In addition, your information will only be used during report writing and not after completing the project.

Voluntary participation
Participation in the study is voluntary. You can withdraw your consent to participate in the study at any time and without stating any particular reason. This will not have any consequences for your further treatment. If you wish to participate, sign the declaration of consent on the final page. If you agree to participate at this time, you may later on withdraw your consent without your treatment being affected in any way. If you later on wish to withdraw your consent or have questions concerning the study, you may contact:

Berhane Megerssa Ereso
Mobile phone number +251917804469
Email address – berhanemegerssa2004@gmail.com
Jimma University, Ethiopia

Supervisors

1. Mette Sagbakken (PhD, Associate professor)

Email address mette.sagbakken@nakmi.no
Mobile phone number +4741576964

2. Solomon Yimer (PhD, Postdoc)

Email address yimsolo@yahoo.com
Mobile phone number +4747687670

Releasing material and data to other parties
If you agree to participate in the study, you also consent de-identified data being released to local and regional health offices in Ethiopia.

Right to access and right to delete your data
If you agree to participate in the study, you are entitled to have access to what information is registered about you. You are further entitled to correct any mistakes in the information we have registered. If you withdraw from the study, you are entitled to demand that the collected data are deleted, unless the data have already been incorporated in analyses or used in scientific publications.

Funding and the role of Strategic and Collaborative Capacity Development in Ethiopia and Africa (SACCADE) Project
The study will be supported by the Strategic and Collaborative Capacity Development in an Ethiopian and African (SACCADE) project, University of Oslo. The SACCADE project will

cover expenses related with personal, materials/ supplies and transportation. It does not have any conflict of interest with any other project

Information about the outcome of the study

You are fully entitled to receive information about the result and outcome of this study.

Consent for participation in the study

I am willing to participate in the study.

(Signature of the study participant, date)

I confirm that I have given information about the study.

(Signature of the data collector, date)

1. Interview guide for Zonal /Woreda health office head and TB program coordinator/supervisors of health extension workers

Background information

Sex ------------- Age -------------------- Profession --------------------------

Responsibility on the program --

Training on TB DOTS ---------------------------- If yes, how long? --------------------------

Introductory/open questions:

1. Would you please tell me about your experience with the national TB control program?

2. Can you please tell me what resources or elements of the treatment program that has to be present to ensure good quality in implementing DOTS?

Theme I. Infrastructure and other resources

1. Do you think that this Zone/Woreda has the necessary infrastructure and other resources to deliver quality DOTS for the community?

Probing questions

- ➢ Would you please mention which infrastructure/resource are absent while necessary for the program?
- ➢ What are the facilitators in relation to infrastructure and other resources?
- ➢ What are the barriers in relation to infrastructure and other resources?

- Has there been an interruption of service due to absence of drug and/or reagents? If so, what measures were taken to solve the interruption?
- In what way do you check the quality of theavailable resource?

Theme II. Compliance to national guideline

2. What are your thoughts on the process/activities performed by health care providers in delivering DOTS for the community in this Zone/ Woreda?

Probing questions

- Do you think that health personnel are performing as per national guideline? (During diagnosis, treatment, follow up, reporting)
- What are possible barriers for compliance of health care providers to the national guideline?
- What are possible facilitators for compliance of health care providers to the national guideline?
- How do you check the quality of the health care providers' performance?
- Have you conducted 'supportive supervision' in this year? How frequent? Was feedback provided in written/orally?

Theme III. Treatment adherence and outcome

3. Can you please tell me about your thoughts and experience in regards to TB treatment adherence in general in this Zone/Woreda?
4. Can you please tell me about your thoughts and experience in regards to the treatment outcome in this Zone/Woreda?

Probing questions

- What are possible explanations/causes for patients' not adhering to treatment?
- What are possible facilitators for patients in completing their treatment?
- What are possible barriers for patients in completing their treatment?
- What are the possible causes for the present (poor/good) TB treatment outcomes?
- What are the possible facilitators and barriers for favorable treatment outcomes?

5. What do you think about facility based versus community based DOTS in this Zone/Woreda? Are both implemented?

6. In your experience, are there any differences in regards to e.g. quality or effectiveness between these? Would you please explain? What suggestions would you give to improve the existing TB control program?
7. Do you have any other information or comments you would like to provide regarding the DOTS strategy?

2. Interview guide for DOTS provider

DOTS site _________________________

Background information

Sex ------------- Age -------------------- Profession ---------------------------
Responsibility in the program ---

Training on TB DOTS ----------------------------- If yes, how long? --------------------------

Introductory /open questions:

1. Would you please tell me about your experience in regards to the national TB control program?

2. Can you please tell me what resources or elements of the treatment program have to be present to ensure good quality in implementing DOTS?

Theme I. Infrastructure and other resources

3. Do you think that this Zone/Woreda has the necessary infrastructure and other resources to deliver quality DOTS for the community?

Probing questions

- Would you please mention which infrastructure/resource are absent while necessary for the program?
- What are the facilitators in relation to infrastructure and other resources?
- What are the barriers in relation to infrastructure and other resources?
- Has there been an interruption of service due to absence of drug and/or reagents? If so, what measures were taken to solve the interruption?

Theme II. Compliance to national guideline

4. What are your thoughts on the process/activities that you have been providing for the community in this DOTS site?

Probing questions

- Do you think that you and your colleagues are performing according to the national guideline? (During diagnosis, treatment, follow up, reporting). Why? Why not?
- What are possible barriers for you to provide DOTS according to the national guidelines?
- What are possible facilitators for you to provide DOTS according to the national guidelines?
- Have you received any type of supervision for performing TB activities? How frequent? Have you received feedback in written/orally? Was it useful?

Theme III. Treatment adherence and outcome

5. Can you please tell me about your thoughts and experience in regards to TB treatment adherence in general in this Zone/Woreda?

6. Can you please tell me about your thoughts and experience in regards to the treatment outcome in this Zone/Woreda?

Probing questions

- What are possible explanations/causes for patients' not adhering to treatment?
- What are possible facilitators for patients in completing their treatment?
- What are possible barriers for patients in completing their treatment?
- What are the possible causes for the present (poor/good) TB treatment outcomes?
- What are the possible facilitators and barriers for favorable treatment outcomes?

7. What do you think about facility based versus community based DOTS in this Zone/Woreda? Are both implemented?

8. In your experience, are there any differences in regards to e.g. quality or effectiveness between these? Would you please explain? What suggestions would you give to improve the existing TB control program?

9. Do you have any other information or comments you would like to provide regarding the DOTS strategy?

3. Interview guide for Regional Health Bureau Head/ Program manager

Background information

Sex ------------ Age ------------------- Profession -------------------------

Responsibility on the program ---

Training on TB DOTS --------------------------- If yes, how long? -------------------------

Introductory /open question:

1. Would you please tell me about your experience in regards to the national TB control program?

Theme I. Infrastructure and other resources

2. What do you think about the availability of necessary infrastructure and other resources for implementation of TB DOTS in Oromia region?

Probing questions

- How did you see the distribution pattern for all Zones under Oromia region?
- Would you please tell me about the source for fund/resources?
- Would you please mention which infrastructure/resource are absent while necessary for the program?
- What are the facilitators in relation to infrastructure and other resources?
- What are the barriers in relation to infrastructure and other resources?
- How do you see the utilization of allocated resources for DOTS in different Zones? What about Jimma Zone in particular?

Theme II. Compliance to national guideline

3. How do you see the general performance of DOTS in Oromia Region? What about in Jimma Zone?

Probing questions

- Do you think that health personal are performing as per national guideline/as expected? (timely, complete, accurate reporting, etc)
- What do you think are the possible barriers for compliance to the national guideline?

- What do you think are the possible facilitators for compliance to the national guideline?
- Have you conducted supportive supervision this year? How frequent? Was feedback provided in written/orally?

Theme III. DOTS outcome

4. What do you think about the overall performance and outcome of DOTS in Oromia Region? What about in Jimma Zone?

Probing questions

- How did you see the overall performance?
- How do you see the outcome of DOTS? Is it as expected?
- What are the possible explanations/causes for this outcome?
- What do you think are the possible facilitators and barriers for favorable outcome?

5. What do you think about facility based versus community based DOTS in Oromia? Are both implemented?

6. In your experience, are there any differences in regards to e.g. quality or effectiveness between these? If yes, can you explain why such differences exists?

7. What suggestions would you give to improve the existing TB control program?

8. Do you have any other information or comments you would like to provide regarding the DOTS strategy?

4. Interview guide for TB patient

Background information of the respondent

Age --------------------- Sex -------------------------- Educational status --------------------------------

Occupational status -------------------------------------- Residence --------------------------------

DOTS site -- Duration of treatment -----------------------------------

Introductory /open question:

1. Can you tell me about your experience being a TB patient at this treatment site? (The diagnostic process, the daily visits to the treatment site)

Theme I. Infrastructure and other resources

2. What is your impression in regards to how the daily TB treatment is performed?

3. Do you think that there are enough resources? (e.g. human power, equipment for diagnostics tests, supplies and TB medicines)

Probing questions

- Where do you receive treatment? Is it difficult for you to come daily for the treatment? If so, in what way?
- Please tell me more about the organization of treatment and care provided for you (waiting hours, queues, physical condition of DOTS site, walking distance, transport)
- Do you have suggestions regarding how this treatment arrangement (DOTS) could be done to help in improving TB control in the community?
- What changes could be made to help patients getting diagnosed and receiving treatment?
- What changes could be made to help patients complete their TB treatment?
- Do you think that your daily life has been affected? If so, how does this treatment affect your daily life? (Expense, time, work, family/social-life)

Theme II. Compliance to national guideline

4. Are you familiar with how the TB treatment is supposed to be conducted (the national guidelines)

5. Did you think that you have been receiving TB care as recommended in the national guideline?

Probing questions

- Was this DOTS site your first choice when you wanted help? If so, why? If not, where?
- Did you discuss your choice of treatment site with health care provider? If no, why not?
- Does the health care provider/treatment supporter observe you daily when you swallow your drugs? If not, why not?
- Can you tell me about the daily meeting with the health personnel? (Communication, friendly? Room for asking questions? follow-up, mal-treatment)

- Have you learned more about TB, causes of TB, treatment and prevention of TB after you started the treatment? Who/ what are your source of information?
- So far what are the major problems you have been faced in receiving TB care?

Theme III. Treatment adherence and outcome

6. What do you think is the first symptoms of TB?

7. Do you think that the treatment you are offered here is the right (sufficient) treatment for TB?

8. At what point would you think that you have recovered from TB?

Probing questions

- What can you say about your daily TB treatment? Have you ever interrupted your treatment? If so, why? For how long?
- What do you think about the consequence of interrupting the TB treatment?
- Would you please explain the possible facilitators and barriers for favorable outcome of TB treatment?

9. What do you think about TB treatment being offered in the health facility versus being offered in the community? (Researcher must provide examples of these types of organization)

10. Do you think there could be important differences and similarities between these ways of organizing treatment?

11. What suggestions would you give to improve the existing TB treatment?

12. Do you have any other information or comments regarding the existing TB care?

Afan oromo version of information sheets and consent forms

1. **Gaaffii hirmaannaa qorannoo pirojektii qindeessitoota/hojii gaggeesitoota sagantaa w/ra fayyaa naannoo fi godinaa**

Raawwannaa fi qulqullina Tarsiimoo Koorsii gabaabaa yaaliinsa daawwannaa kallaattii dhukkuba “Tiibii’’ (DOTS) godina jimmaa, kibba lixa itiyoophiyaa.

Seensa

Maqaan koo---------------- -------------Yunivarstii osloo,Noorwayitti ani baratаа PhD ti.

Sababa raawwatiinsa naannoo sagantaa to’achuufi Ittisuu Tiibii irraatti muuxxaannoo qabdaniifi qo’annaa qorannoo irraatti akka hirmaattan kabajaan isin affeerreera.Tarreen karoora qorannoo kanaa ragaa kana irraatti tarreeffameera.Qorannoon kun maaliifi akka gaggeeffamu,maal of keessaa akka qabu, baruun isiniif barbaachisaa dha. Wixinee qorannoo irraatti hirmaachuuf murteessuu keessaaniin dura odeeffannoo kana of eeggannoon hubachuu fi waliigala isaa dubbisuuf adaraa yeroo kennaa! Wanti ifa isiniif hin taane yoo jiraate ykn Odeeffannoo dabalataa argachuuf gaafa dhaa.

Faayidaafi duub jalee

Dhikkubni “Tiibii dhukkuba daddarboo dha,akka waliigalaatti umrii kamiyyuu irraatti saala lamaanuu du’aafis ta’e dhukkuba cimaafi kan nama saaxilu dha.Dhukkubni “Tiibii godina jimmaa dabalatee itiyoophiyaa keessaatti beekamaa dha. Haala fudhatama,danda’amaafi baasii xiqqaa ta’een dhukkubicha to’achuun barbaachisaa dha. Yaadni piroojektii qorannoo kanaas;-itiyoophiyaa keessaa godina jimmaatti raawwii fi qulqullina waliigalaa tarsiimoo koorsii gabaabaa yaalinsa daawwannaa kallattii/’Tiibii’-DOTS/ sakattaa’uu dha. Beekumsi qorannoo kana irraa argamu raawwiifi qulqullina sagantichaa foyyeessuuf odeeffaannoo gahaa kennuuf kan isin gargaaru dha. Aangeeffamaa Qindeessaa ykn hojii gaggeessaa w/ra fayyaa ta’uu keessaniin sochiiwwan sagantaa ‘Tiibii’waliigalaa baayyee irraatti waan hirmaattaniifi saganticha haala itti raawwatu irraatti beekumsa hedduu akka qabdanu ni amanna.Kanaafi qorannocha keenyaaf beekumsa barbaachisaa kennuu akka dadeessanu hubannee itti yaaduun isin filanneerra.

Qorannochi Maal qabata?

Qorannoo kana keessaatti waa’ee koorsii gabaabaa yaalinsa daawwannaa kallaattii ‘Tiibii’ muuxannoofi ilaalcha qabdanu irraatti ani gaaffii isin gaafadha. Gaaffifi deebiichi sa’aatii tokko kan fudhatu yoo ta’u yeroonifi bakki isiniif mijaa’aa ta’etti kan ta’u dha. Hirmaachuufi eeyyamaa yoo taatan gaaffifi deebichi Teeppiidhaan kan waraabamu ta’a. Waa’ee sagantaa to’annoo ‘’Tiibii’’ fi sagantaa kana yeroo raawwachistan jiraachuu wantoota mijeessitootaafi danqarsoota addaan baasuu irraatti gaaffii muraasa isin gaafachuu nan barbaada.Yoo hirmaattanis ta’e dhiistan haala kamiinuu aageeffama keessan irraatti dhiibbaa hin qabu.

Jiraachuu Faayidaa fi miidhaa

Waa'ee Tarsiimoo 'Tiibii'DOTS odeffaannoo nuuf kennitanu gara sa'aatii tokko isin jalaa gubuu ni danda'a. Qorannoo kana irraatti hirmaachuu keessaaniifi kallattidhaan faayidaan isin argattan hin jiru ta'a garuu amanamummaafi bilisaan deebiin kennitan foyya'insa raawwii fi qulqullina tajaajila 'Tiibii'wojin walqabateef odeeffannoo gabbifachuufi gahee qaba.

Odeeffaannoo kennitan maal ta'uu danda'a?

Ragaan waa'ee keessan galmaa'ee kun faayidaa qorannoo kanaafi qofa oola. Ragaan kun kan raawwatu maqaafi lakkofsa eenyummaa kallaattiin haala odeeffaannoo hin beekamneeni dha. Lakkofsa addaa ragaa keessan isiniin wal qabsiisu ni fayyadamna,akkasumas odeeffaannoon isin nuuf kennitanu icitiin ni qabama,Ragaan hundu saanduqa keessatti furtuun kan qabamuufi lakkofsa icitii kompurataan ni eegama. Dabalataanis odeeffaannoon keessan kan fayyadamnu piroojektii eega xumurree booda osoo hin ta'in yeroo gabaasni barreeffamu qofa dha.

Hirmaannaa Fedhiinii

Hirmaanaan qorannoo kana keessaatti gaggeffamu fedhii irraatti kan hundaa'ee dha.Sababa dhuunfaa kamiyyuu osoo hin dhiheessin yeroo barbaaddan keessaatti fedhii keessan haquu ni dandeessu.Kunis yaalinsa itti fufu irraatti miidhaa wanta tokko isin irraatti hin qaqqabsiisu.hirmaachuu kan barbaaddan yoo ta'e fuula xumuraa waliigaltee irraatti mallateessaa.Yeroo kana keessaatti hirmaachuufi yoo waliigaltan, karaa kamiinuu yaalinsa keessan haala hin miineen booda irraatti waliigaltee keessan haquu ni dandeessu. Booda irraatti waliigaltee keessan haquu yoo barbaaddan ykn gaaffii qorannocha ilaallatu yoo qabaattan:

Birhaanee magarsaa Irreessoo

Lakkofsa mobaayilii 0917804469

Teessoo imeelii berhanemegerssa2004@gmail.com

Yunivarstii jimmaa, itiyoophiyaa

Supparvaayizaroota

1. Mette Sagbakken(PhD, pirofeesara asoosheetii)
   Teessoo imeelii mette.sagbkken@nakmi.no

   Lakkofsa mobaayilii +4741576964

2. Salamoon Yimar(PhD,postdoc)
   Teessoo imeelii yimsolo@yahoo.com
   Lakkofsa mobaayilii +4747687670

Argachu ykn dubbisu dandeessu

Miseensota kan birootiif ragaafi meeshaalee dabarsuu

Qorannoocha irraatti hirmaachuufi hanga waliigaltanitti ragaa eenyummaa hin ibsine itiyoophiyaatti w/ra fayyaa naannoofi godina keessaatti ragaa darbuufi eeyyamtanittu jechuu dha.

Mirga argachuufi haquu ragaa

Qorannocha irraatti hirmaachuuf hanga waliigaltanitti waa'ee keessanii odeeffannoo galmaa'ee argachuu ni dandeessu. Odeeffaannoo nuti galmeessine keessaatti dogongora kamiyyuu dabalataan sirreessuu ni dandeessu.Qorannocha keessaa bahuu yoo barbaaddan; hanga ragichi hin qindoofnetti ykn tajaajila maxxansaafi hin galleetti ragaa sassaabame haquu ni dandeessu.

Maallaqaafi gahee pirojektii tarsiimoo walgargaarsaa misooma dandeetti itiyoophiyaafi afriikaa (SACCADE)

Qo'annochi maallaqaan kan gargaaramu pirojektii tarsiimoo walgargaarsaa misooma dandeetti itiyoophiyaafi afriikaa (SACCADE) irraa qorannoofi kan argameetiini dha.Pirojektiin SACCADE baasiiwwan geejjibaa, namaafi dhiheessa meeshaaleetiin walqabatu ni aguuga.pirojektichi pirojektii kamiyyuu waliin walitti bu'iinsa faayidaa hin qabu.

Odeeffaannoo waa'ee bu'aa qo'annoochaa

Waa'ee xumuraafi bu'aa qo'annochaa odeeffaannoo fudhachuuf mirga guutuu qabdu.

Qo'annocha irraatti hirmaachuufi eeyyamaa ta'u

Ani qo'annocha irraatti hirmaachuufi eeyyameera.

(Mallaattoo hirmaataa qo'annichaa fi guyyaa)

Waa'ee qo'annochaa odeeffaannoo kennuu koo nan mirkaneessa.

Mallaattoo sassaabaa Ragaa fi guyyaa

2. Gaaffii hirmaannaa qorannoo pirojektii kennitoota Koorsii gabaabaa yaaliinsa daawwannaa kallaattii dhukkuba "Tiibii'' (DOTS)

Raawwannaa fi qulqullina Tarsiimoo Koorsii gabaabaa yaaliinsa daawwannaa kallaattii dhukkuba "Tiibii'' (DOTS) godina jimmaa, kibba lixa itiyoophiyaa.

Seensa

Maqaan koo---------------- -------------Yunivarstii osloo,Noorwayitti ani baratatu PhD ti.

Sababa raawwatiinsa naannoo sagantaa to’achuufi Ittisuu Tiibii irraatti muuxxaannoo qabdaniifi qo’annaa qorannoo irraatti akka hirmaattan kabajaan isin affeerreera.Tarreen karoora qorannoo kanaa ragaa kana irraatti tarreeffameera.Qorannoon kun maaliifi akka gaggeeffamu,maal of keessaa akka qabu, baruun isiniif barbaachisaa dha. Wixinee qorannoo irraatti hirmaachuuf murteessuu keessaaniin dura odeeffannoo kana of eeggannoon hubachuu fi waliigala isaa dubbisuuf adaraa yeroo kennaa! Wanti ifa isiniif hin taane yoo jiraate ykn Odeeffannoo dabalataa argachuuf gaafa dhaa.

Faayidaafi duub jalee

Dhikkubni “Tiibii dhukkuba daddarboo dha,akka waliigalaatti umrii kamiyyuu irraatti saala lamaanuu du’aafis ta’e dhukkuba cimaafi kan nama saaxilu dha.Dhukkubni “Tiibii godina jimmaa dabalatee itiyoophiyaa keessaatti beekamaa dha. Haala fudhatama,danda’amaafi baasii xiqqaa ta’een dhukkubicha to’achuun barbaachisaa dha. Yaadni piroojektii qorannoo kanaas;-itiyoophiyaa keessaa godina jimmaatti raawwii fi qulqullina waliigalaa tarsiimoo koorsii gabaabaa yaalinsa daawwannaa kallattii/’Tiibii’-DOTS/ sakattaa’uu dha. Beekumsi qorannoo kana irraa argamu raawwiifi qulqullina sagantichaa foyyeessuuf odeeffaannoo gahaa kennuuf kan isin gargaaru dha. Aangeeffamaa Qindeessaa ykn hojii gaggeessaa w/ra fayyaa ta’uu keessaniin sochiiwwan sagantaa ‘Tiibii’waliigalaa baayyee irraatti waan hirmaattaniifi saganticha haala itti raawwatu irraatti beekumsa hedduu akka qabdanu ni amanna.Kanaafi qorannocha keenyaafi beekumsa barbaachisaa kennuu akka dadeessanu hubannee itti yaaduun isin filanneerra.

Qorannochi Maal qabata?

Qorannoo kana keessaatti waa’ee koorsii gabaabaa yaalinsa daawwannaa kallaattii ‘Tiibii’ muuxannoofi ilaalcha qabdanu irraatti ani gaaffii isin gaafadha. Gaaffifi deebiichi sa’aatii tokko kan fudhatu yoo ta’u yeroonifi bakki isiniif mijaa’aa ta’etti kan ta’u dha. Hirmaachuufi eeyyamaa yoo taatan gaaffifi deebichi Teeppiidhaan kan waraabamu ta’a. Waa’ee sagantaa to’annoo ‘’Tiibii’’ fi sagantaa kana yeroo raawwachistan jiraachuu wantoota mijeessitootaafi danqarsoota addaan baasuu irraatti gaaffii muraasa isin gaafachuu nan barbaada. Yoo hirmaattanis ta’e dhiistan haala kamiinuu aageeffama keessan irraatti dhiibbaa hin qabu.

Jiraachuu Faayidaa fi miidhaa

Waa’ee Tarsiimoo ‘Tiibii’DOTS odeffaannoo nuuf kennitanu gara sa’aatii tokko isin jalaa gubuu ni danda’a. Qorannoo kana irraatti hirmaachuu keessaaniifi kallattidhaan faayidaan isin argattan hin jiru ta’a garuu amanamummaafi bilisaan deebiin kennitan foyya’insa raawwii fi qulqullina tajaajila ‘Tiibii’wojin walqabateef odeeffannoo gabbifachuufi gahee qaba.

Odeeffaannoo kennitan maal ta’uu danda’a?

Ragaan waa’ee keessan galmaa’ee kun faayidaa qorannoo kanaafi qofa oola. Ragaan kun kan raawwatu maqaafi lakkofsa eenyummaa kallaattiin haala odeeffaannoo hin beekamneeni dha.

Lakkofsa addaa ragaa keessan isiniin wal qabsiisu ni fayyadamna, akkasumas odeeffaannoon isin nuuf kennitanu icitiin ni qabama,Ragaan hundu saanduqa keessatti furtuun kan qabamuufi lakkofsa icitii kompurataan ni eegama. Dabalataanis odeeffaannoon keessan kan fayyadamnu piroojektii eega xumurree booda osoo hin ta'in yeroo gabaasni barreeffamu qofa dha.

Hirmaannaa Fedhiinii

Hirmaanaan qorannoo kana keessaatti gaggeffamu fedhii irraatti kan hundaa'ee dha.Sababa dhuunfaa kamiyyuu osoo hin dhiheessin yeroo barbaaddan keessaatti fedhii keessan haquu ni dandeessu.Kunis yaalinsa itti fufu irraatti miidhaa wanta tokko isin irraatti hin qaqqabsiisu.hirmaachuu kan barbaaddan yoo ta'e fuula xumuraa waliigaltee irraatti mallateessaa.Yeroo kana keessaatti hirmaachuufi yoo waliigaltan, karaa kamiinuu yaalinsa keessan haala hin miineen booda irraatti waliigaltee keessan haquu ni dandeessu. Booda irraatti waliigaltee keessan haquu yoo barbaaddan ykn gaaffii qorannocha ilaallatu yoo qabaattan:

Birhaanee magarsaa Irreessoo

Lakkofsa mobaayilii 0917804469

Teessoo imeelii berhanemegerssa2004@gmail.com

Yunivarstii jimmaa, itiyoophiyaa

Supparvaayizaroota

1. Mette Sagbakken(PhD, pirofeesara asoosheetii)
Teessoo imeelii mette.sagbkken@nakmi.no

   Lakkofsa mobaayilii +4741576964

2. Salamoon Yimar(PhD,postdoc)
Teessoo imeelii yimsolo@yahoo.com
Lakkofsa mobaayilii +4747687670

   Argachu ykn dubbisu dandeessu.

Miseensota kan birootiif ragaafi meeshaalee dabarsuu

Qorannoocha irraatti hirmaachuufi hanga waliigaltanitti ragaa eenyummaa hin ibsine itiyoophiyaatti w/ra fayyaa naannoofi godina keessaatti ragaa darbuufi eeyyamtanittu jechuu dha.

Mirga argachuufi haquu ragaa

Qorannocha irraatti hirmaachuuf hanga waliigaltanitti waa'ee keessanii odeeffannoo galmaa'ee argachuu ni dandeessu. Odeeffaannoo nuti galmeessine keessaatti dogongora kamiyyuu dabalataan sirreessuu ni dandeessu.Qorannocha keessaa bahuu yoo barbaaddan; hanga ragichi hin qindoofnetti ykn tajaajila maxxansaafi hin galleetti ragaa sassaabame haquu ni dandeessu.

Maallaqaafi gahee pirojektii tarsiimoo walgargaarsaa misooma dandeetti itiyoophiyaafi afriikaa (SACCADE)

Qo'annochi maallaqaan kan gargaaramu pirojektii tarsiimoo walgargaarsaa misooma dandeetti itiyoophiyaafi afriikaa (SACCADE) irraa qorannoofi kan argameetiini dha.Pirojektiin SACCADE baasiiwwan geejjibaa,namaafi dhiheessa meeshaaleetiin walqabatu ni aguuga.pirojektichi pirojektii kamiyyuu waliin walitti bu'iinsa faayidaa hin qabu.

Odeeffaannoo waa'ee bu'aa qo'annoochaa

Waa'ee xumuraafi bu'aa qo'annochaa odeeffaannoo fudhachuuf mirga guutuu qabdu.

Qo'annocha irraatti hirmaachuufi eeyyamaa ta'u

Ani qo'annocha irraatti hirmaachuufi eeyyameera.

(Mallaattoo hirmaataa qo'annichaa fi guyyaa)

Waa'ee qo'annochaa odeeffaannoo kennuu koo nan mirkaneessa.

Mallaattoo sassaabaa Ragaa fi guyyaa

3. **Gaaffii hirmaannaa qorannoo pirojektii dhukubsattoota 'Tiibiitiif' gaaffiifi deebii gadi fageenyaatiif)**
   Raawwannaa fi qulqullina Tarsiimoo Koorsii gabaabaa yaaliinsa daawwannaa kallaattii dhukkuba "Tiibii'' (DOTS) godina jimmaa, kibba lixa itiyoophiyaa.

<u>Seensa</u>

Maqaan koo---------------- -------------Yunivarstii osloo,Noorwayitti ani baratuPhD ti.

Sababa dhukkuba 'Tiibii' jedhamee beekamu isin irratti waan argameefi hirmaannaa qo'annaa qorannoo irraatti akka hirmaattan kabajaan isin affeerreera.

Tarreen karoora qorannoo kanaa ragaa kana irraatti tarreeffameera.Qorannoon kun maaliifi akka gaggeeffamu, maal of keessaa akka qabu, baruun isiniif barbaachisaa dha. Wixinee qorannoo irraatti hirmaachuuf murteessuu keessaaniin dura odeeffannoo kana of eeggannoon hubachuu fi waliigala isaa dubbisuuf adaraa yeroo kennaa! Wanti ifa isiniif hin taane yoo jiraate ykn Odeeffannoo dabalataa argachuuf gaafa dhaa.

Faayidaafi duub jalee

Dhikkubni “Tiibii dhukkuba daddarboo dha,akka waliigalaatti umrii kamiyyuu irraatti saala lamaanuu du’aafis ta’e dhukkuba cimaafi kan nama saaxilu dha.Dhukkubni “Tiibii godina jimmaa dabalatee itiyoophiyaa keessaatti beekamaa dha. Haala fudhatama,danda’amaafi baasii xiqqaa ta’een dhukkubicha to’achuun barbaachisaa dha. Yaadni piroojektii qorannoo kanaas;- itiyoophiyaa keessaa godina jimmaatti raawwii fi qulqullina waliigalaa tarsiimoo koorsii gabaabaa yaalinsa daawwannaa kallattii/’Tiibii’-DOTS/ sakattaa’uu dha.

Miseensotni hawaasaa tajaajila gaarii akka argataniifi raawwiin fi qulqullina sagantichaa foyyeessuufi jecha qorannaa kana irraa beekumsi argamu odeeffannoo faayidaa qabeessa ta’e akka murtii kennitootaafi kenninu kan nu gargaaru dha.

Kana hojjachuu akka dandeenyuufi waa’ee dhukkubsattoota ‘Tiibii’ yaalinsa irra jiranii muuxxaannoofi beekumsa keessaan argachuun baayyee barbaachisaa dha. Kanaafi qorannocha keenyaafi beekumsa barbaachisaa kennuu akka dandeessanu hubannee itti yaaduun isin filanneerra.

Qorannochi Maal qabata?

Yaalinsa fudhataa hanga taatanitti qorannoo kana keessaatti waa’ee koorsii gabaabaa yaalinsa daawwannaa kallaattii ‘Tiibii’ gaaffifi deebii isniif gochuu nan barbaada. Muuxannoof ilaalcha kunuunsa ‘Tiibii’ irraatti qabdanu fi yeroo kunuunsa ‘Tiibii’ mijeessitootaafi jiraachuu rakkoo addaan baasuuf gaaffii muraasa isin gaafachuu nan barbaada. Gaaffifi deebiichi sa’aatii tokko kan fudhatu yoo ta’u yeroonifi bakki isiniif mijaa’aa ta’etti kan ta’u dha. Hirmaachuufi eeyyamaa yoo taatan gaaffifi deebichi Teeppiidhaan kan waraabamu ta’a. Qorannoo kana irraatti hirmaattanis dhiistanis yaalinsa argattan irraatti dhibbaa kamuu hin qabu.Yaalinsa Kanaan dura argattan itti fufaan ni fudhattu.

Jiraachuu Faayidaa fi miidhaa

Odeeffaannoo waa’ee argannoo ‘Tiibii’ fi yaalinsa isaa nuuf kennitan humnaafi yeroo keessan isa mi’awaa sa’aa tokko kan ta’u isin jalaa gubuu ni danda’a. Qorannoo kana irraatti hirmaachuu keessaaniifi kallattidhaan faayidaan isin argattan hin jiru ta’a garuu amanamummaafi bilisaan deebiin kennitan foyya’insa raawwii fi qulqullina tajaajila ‘Tiibii’wojin walqabateef odeeffannoo gabbifachuufi gahee qaba.

Odeeffaannoo kennitan maal ta’uu danda’a?

Ragaan waa’ee keessan galmaa’ee kun faayidaa qorannoo kanaafi qofa oola. Ragaan kun kan raawwatu maqaafi lakkofsa eenyummaa kallaattiin haala odeeffaannoo hin beekamneeni dha. Lakkofsa addaa ragaa keessan isiniin wal qabsiisu ni fayyadamna, akkasumas odeeffaannoon isin nuuf kennitanu icitiin ni qabama,Ragaan hundu saanduqa keessatti furtuun kan qabamuufi

lakkofsa icitii kompurataan ni eegama. Dabalataanis odeeffaannoon keessan kan fayyadamnu piroojektii eega xumurree booda osoo hin ta'in yeroo gabaasni barreeffamu qofa dha.

Hirmaannaa Fedhiinii

Hirmaanaan qorannoo kana keessaatti gaggeffamu fedhii irraatti kan hundaa'ee dha.Sababa dhuunfaa kamiyyuu osoo hin dhiheessin yeroo barbaaddan keessaatti fedhii keessan haquu ni dandeessu.Kunis yaalinsa itti fufu irraatti miidhaa wanta tokko isin irraatti hin qaqqabsiisu.hirmaachuu kan barbaaddan yoo ta'e fuula xumuraa waliigaltee irraatti mallateessaa.Yeroo kana keessaatti hirmaachuufi yoo waliigaltan, karaa kamiinuu yaalinsa keessan haala hin miineen booda irraatti waliigaltee keessan haquu ni dandeessu. Booda irraatti waliigaltee keessan haquu yoo barbaaddan ykn gaaffii qorannocha ilaallatu yoo qabaattan:

Birhaanee magarsaa Irreessoo

Lakkofsa mobaayilii 0917804469

Teessoo imeelii berhanemegerssa2004@gmail.com

Yunivarstii jimmaa, itiyoophiyaa

Supparvaayizaroota

1. Mette Sagbakken(PhD, pirofeesara asoosheetii)
   Teessoo imeelii mette.sagbkken@nakmi.no

   Lakkofsa mobaayilii +4741576964

2. Salamoon Yimar(PhD,postdoc)
   Teessoo imeelii yimsolo@yahoo.com
   Lakkofsa mobaayilii +4747687670
   Argachu ykn dubbisu dandeessu.

Miseensota kan birootiif ragaafi meeshaalee dabarsuu

Qorannoocha irraatti hirmaachuufi hanga waliigaltanitti ragaa eenyummaa hin ibsine itiyoophiyaatti w/ra fayyaa naannoofi godina keessaatti ragaa darbuufi eeyyamtanittu jechuu dha.

Mirga argachuufi haquu ragaa

Qorannocha irraatti hirmaachuuf hanga waliigaltanitti waa'ee keessanii odeeffannoo galmaa'ee argachuu ni dandeessu. Odeeffaannoo nuti galmeessine keessaatti dogongora kamiyyuu dabalataan sirreessuu ni dandeessu.Qorannocha keessaa bahuu yoo barbaaddan; hanga ragichi hin qindoofnetti ykn tajaajila maxxansaafi hin galleetti ragaa sassaabame haquu ni dandeessu.

Maallaqaafi gahee pirojektii tarsiimoo walgargaarsaa misooma dandeetti itiyoophiyaafi afriikaa (SACCADE)

Qo'annochi maallaqaan kan gargaaramu pirojektii tarsiimoo walgargaarsaa misooma dandeetti itiyoophiyaafi afriikaa (SACCADE) irraa qorannoofi kan argameetiini dha.Pirojektiin SACCADE baasiiwwan geejjibaa,namaafi dhiheessa meeshaaleetiin walqabatu ni aguuga.pirojektichi pirojektii kamiyyuu waliin walitti bu'iinsa faayidaa hin qabu.

Odeeffaannoo waa'ee bu'aa qo'annoochaa

Waa'ee xumuraafi bu'aa qo'annochaa odeeffaannoo fudhachuuf mirga guutuu qabdu.

Qo'annocha irraatti hirmaachuufi eeyyamaa ta'u

Ani qo'annocha irraatti hirmaachuufi eeyyameera.

(Mallaattoo hirmaataa qo'annichaa fi guyyaa)

Waa'ee qo'annochaa odeeffaannoo kennuu koo nan mirkaneessa.

Mallaattoo sassaabaa Ragaa fi guyyaa

Afan oromo version of interview guides

1. Agarsiisa Gaaffii fi deebii godinaa,gaggeessaa waajjira fayyaa aanaa, qindeessaa sagantaa ‘Tiibii’/supparvaayizarii hojjataa eksiteeshinii Fayyaa

Odeeffannoo Dub jalee

Saalaa ----------------umurii -------------------- ogummaa -------------------------sanganticha irratti- itti gaafatamummaa ---------------------leenjii ‘Tiibii’ DOTS------------------------------yoo eeyyee ta’e , yeroo hangamiif?------------------------------------

Seensaa/Gaaffii galumsaa

1. Maaloo, sagantaa to’annaa biyyooleessaa ‘Tiibii’ Muuxannoo keessaan natti himtaa?

2. Qulqullinaan hojii irra ooluu DOTS haal gaariin mirkaneessuuf saganataa yaalinsaafi qabeenya ykn wantoota barbaachisan dhiyaachuu qaban natti himuu dandeessuu?

Dhimma 1. Bu`uuralee misoomaa fi qabeenyaa biroo

3. Godinni/aanaan kun bu’uuralee misoomaa fi qabeenya biroo barbaachisaa ‘DOTS’ qulqullina qabuhaawaasaaf kennu qaba jettanii yaadduu ?

Gaaffilee kaka’umsaa

- Bu’uuralee misoomaa/ qabeenya sagantichaaf barbaachisaa ta’anii amma kan hin jirre maaloo kami akka ta’e tarreessuu dandeessuu?
- bu`uuralee misoomaa fi qabeenyaa biroon walqabate haala mijeessitootni kam faadha?
- Bu`uraalee misoomaa fi qabeenya biroon walqabtan haalli rakkisoon kam faadha?
- Sababa Dhabamuu qorichaatiin yookiin keemikaala laabiraatooriitiin tajaajilli dhaabbatee jiraa? Eeyyee yoo ta’e tarkaanfilee maaltu fudhatame rakkoo tajaajila addan kute hiikuuf ?
- Qulqullina qabeenya jiranii haala kamiin mirkaneessitu?

Dhimma 2. Akkaataa agarsiisa biyyooleessaan hojjachuu

4. Hawaasaa godinaa /aanaa kanaaf kenninsa ‘DOTS ‘ irratti adeemsa /gochaalee kennitoota kunuunsa fayyaan hojjatame ilaalchisee yaadni keessaan maali?

Gaffilee kaka’umsaa

- Namoonni fayyaa irratti hojjatan akkaatadhuma agarsiisa biyooleessaatiin hojjataa jiru jettani yaadduu? (yeroo argama dhukkubaa ,yaalinsa,hordoffii ,gabaasa)
- Agarsiisa biyyooleessaatiin hojii irraa oolmaa kunuunsa fayyaa kennitootaaf rakkooleen jiran maal faadha?
- Agarsiisa biyyooleessaatiin hojiirra oolmaaa kunuunsa fayyaa kennitootaaf haala mijeessitoota kamfaa dhaa ?

- Raawwatiinsa qulqullina keenitoota kunuunsa Fayyaa akkamiin mirkaneessitu?
- Bara kana to`annoo gargaarsaa rawwattee beektaa? yeroo hagamiif? Dub-deebiin bareeffamaan/afaaniin jira turee?

Dhimmaa 3ffaa Yaalinsa waliirra hin cinnee fi bu`aa

5. Godinaa/aanaa kana keessaatti Yaalinsa 'Tiibii' walirraa hin cinne waliigala ilaalchisee muuxannoo fi yaada keessan maaloo natti himuu dandeessuu?

6. Godinaa/aanaa kana keessaatti bu'aa yaalinsa 'Tiibii' ilaalchisee muuxannoo fi yaada keessan maaloo natti himuu dandeessuu?

Gaaffilee kaka`umsaa

- Dhukkabsattootni Sababni /ibsamni yaalinsa hordofanii akka hin fudhanne maalfaa ta`u danda`a?
- Dhukkubsattootni yaalinsa isaanii akka xumurraniif haala mijeessiitoota maal fa'i ta'uu danda'uu ?
- Dhukkubsattootni yaalinsa isaanii akka hin xumurreef rakkoowwan jiran maalfaa ta'uu danda'uu?
- Sababooleen Bu'aalee yaalinsaa 'Tiibii' yeroo amma (gaarii/yaraa/ maal ta`u danda`u?
- Bu'aalee Yaalinsaa mijaawaafi rakkoowwaniifi haala mijeessitootni maal faa'i ta`uu danda`a ?

7. Godina /aanaa kanatti 'DOTS'iin haawaasa bu`uureeffatee fi dhaabbata bu`ureeffate jidduu garaagarummaan isaanii maal yaaddu ? Lamaanuu hojiiatamaa jiruu?

8. Muxannoo keessaan keessatti garaagarummaa ilaalachisee fakkeenyaaf Qulqullina yookiin milkaa'inaa jidduu jaraa jiraayi ?

9. Sagantaa To'annaa 'Tiibii' amma jiru fayyeessuuf yaadota maali kennitu?

10. Tarsiimoo 'DOTS' ilaachisee yaadawwan ykn odeeffaannoo biroo kamiyyuu kennuu barbaaddan qaduuyii?

2. Agarsiisa Gaaffii fi deebii kennitoota DOTS iif

Iddoo 'DOTS' --------------------------------

Odeeffannoo Dub jalee

Saalaa -----------------umurii ---------------------ogummaa --------------------------sanganticha irratti- itti gaafatamummaa ----------------------leenjii 'Tiibii' DOTS--------------------------------yoo eeyyee ta'e , yeroo hangamiif?-------------------------------------

Seensaa/Gaaffii galumsaa

1. Maaloo, sagantaa to'annaa biyyooleessaa 'Tiibii' Muuxannoo keessaan natti himtuu ?

2. Qulqullinaan hojii irra ooluu DOTS haal gaariin mirkaneessuuf saganataa yaalinsaafi qabeenya ykn wantoota barbaachisan dhiyaachuu qaban natti himuu dandeessuu?

Dhimma 1. Bu`uuralee misoomaa fi qabeenyaa biroo

3. Godinni/aanaan kun bu'uuralee misoomaa fi qabeenya biroo barbaachisaa 'DOTS' qulqullina qabu haawaasaaf kennu qaba jettanii yaadduu ?

Gaaffilee kaka'umsaa

- Bu'uuralee misoomaa/ qabeenya sagantichaaf barbaachisaa ta'anii amma kan hin jirre maaloo kami akka ta'e tarreessuu dandeessuu?
- bu`uuralee misoomaa fi qabeenyaa biroon walqabate haala mijeessitootni kam faadha?
- Bu`uraalee misoomaa fi qabeenya biroon walqabtan haalli rakkisoon kam faadha?
- Sababa Dhabamuu qorichaatiin yookiin keemikaala laabiraatooriitiin tajaajilli dhaabbatee jiraa? Eeyyee yoo ta'e tarkaanfilee maaltu fudhatame rakkoo tajaajila addan kute hiikuuf ?

Dhimma 2.Akkaataa agarsiisa biyyooleessaan hojjachuu

4. Iddoo 'DOTS ' kana irratti adeemsa /gochaalee hawaasaaf kennaa turtan ilaalchisee yaadni keessaan maali?

Gaffilee kaka'umsaa

- Isiniifi hiriyootni keessan akkaatadhuma agarsiisa biyooleessaatiin hojjataa jirra jettani yaadduu? (yeroo argama dhukkubaa ,yaalinsa,hordoffii ,gabaasa)maaliif/maaliif hin taane?
- Akkaataa agarsiisa biyyoolessaattin DOTS kennuuf rakkooleen jiran maal faadha?
- Akkaataa agarsiisa biyyoolessaattin DOTS kennuuf haala mijeessitoota maalfaa dha ?
- Raawwachiisa gochaalee Tiibiitiif supparviizyinii gosa kamiyyuu fudhattan jiraayii ?yeroo hagamiifi?duub deebii barreeffamaa/afaaniin fudhattaniittuu ?isin fayyadee turee?

Dhimmaa 3ffaa Yaalinsa waliirra hin cinnee fi bu`aa

5. Godinaa/aanaa kana keessaatti Yaalinsa 'Tiibii' walirraa hin cinne waliigala ilaalchisee muuxannoo fi yaada keessan maaloo natti himuu dandeessuu?

6. Godinaa/aanaa kana keessaatti bu'aa yaalinsa 'Tiibii' ilaalchisee muuxannoo fi yaada keessan maaloo natti himuu dandeessuu?

Gaaffilee kaka`umsaa

- Dhukkabsattootni Sababni /ibsamni yaalinsa hordofanii akka hin fudhanne maalfaa ta`u danda`a?
- Dhukkubsattootni yaalinsa isaanii akka xumurraniif haala mijeessiitoota maal fa'i ta'uu danda'uu ?
- Dhukkubsattootni yaalinsa isaanii akka hin xumurreef rakkoowwan jiran maalfaa ta'uu danda'uu?

- Sababooleen Bu’aalee yaalinsaa ‘Tiibii’ yeroo amma (gaarii/yaraa/ maal ta`u danda`u?
- Bu’aalee Yaalinsaa mijaawaafi rakkoowwaniifi haala mijeessitootni maal faa’i ta`uu danda`a ?

7. Godina /aanaa kanatti ‘DOTS’iin haawaasa bu`uureeffatee fi dhaabbata bu`ureeffate jidduu garaagarummaan isaanii maal yaaddu ? Lamaanuu hojiiatamaa jiruu?

8. Muxannoo keessaan keessatti garaagarummaa ilaalachisee fakkeenyaaf Qulqullina yookiin milkaa’inaa jidduu jaraa jiraayi ?

9. Sagantaa To’annaa ‘Tiibii’ amma jiru fayyeessuuf yaadota maali kennitu?

10. Tarsiimoo ‘DOTS’ ilaachisee yaadawwan ykn odeeffaannoo biroo kamiyyuu kennuu barbaaddan qaduuyii?

3. Agarsiisa Gaaffii fi deebii dhukkubsattoota ‘Tiibii’tiif

Odeeffannoo Dub jalee

Saalaa -----------------umurii ---------------------haala barumsaa ----------------------------

Haala hojii--iddoo jireenyaa ---------------------------

iddoo DOTS----------------------------------hanga yeroo yaalinsaa ------------------------

Seensaa/Gaaffii galumsaa

1. Akka dhukkubsataa Tiibii iddoo yaalinsa kanaa taatanitti muuxannoo keessaan natti himuu dandeessuu? (adeemsa argannoo ,deddeebi’iinsa guyyaanii iddoo yaalinsaa)

Dhimma 1. Bu`uuralee misoomaa fi qabeenyaa biroo

2. Haala raawwii yaalinsa Tiibii guyyaa guyyaanii ilaalchisee ilaalchi keessan maali?

3. Qabeenyawwan gahaa jira jettanii yaadduu ?(fakkeenyaafi,humna namaa,meeshaa qorannoo argachuu dhukkubaa,meeshaalee yaalinsaa dabalataa fi qorichaalee ‘ Tiibii’)

Gaaffii kaka’umsaa

- Yaalinsa eessaa fudhattu? Guyyaa guyyaan yaalinsaaf dhufuun isin rakkisaa? Yoo ta’e haala kamiin ?
- Haala qindeessa kunuunsaafi yaalinsaa siif kennamaa ture dabalataan maaloo natti himaa (sa`atii eegumsaa,dabaree, haala qabatama iddoo DOTS, fageenya deemsaan,geejjiba)
- To’annaa ‘Tiibii’ hawaasa keessaa foyyeessuuf kan gargaaru sirni yaalinsaa (DOTS) hojjatame ilaalchisee yaanni keessaan maali?
- Dhukkubsattota dhukkubni isaanii argameefi fi yaalinsa fudhataa jiran gargaruuf jijjiramoota maaliitu raawwatamee jira?

- Dhukkubsattota yaalinsa isaanii akka xumuraniif gargaruuf jijjiramoota maaliitu raawwatamee jira?
- Jireenyi guyyaa guyyaa kee irraatti dhiibbaa uumeera jette yaaddaa? Yoo ta`e akkamitti yaalinsi kun jieenya guyyaa guyyaa kee diibe?(baasii,yeroo,hojii, maatii/jireenya hawaasummaa)

Dhimma 2.Akkaataa agarsiisa biyyooleessaan hojjachuu

4. Yaalinsi 'Tiibii' akkamiin akka kennamuu qabu hubannoo gahaa qabdaayii? (agarsiisaa biyyoolessaa)

5. Kunuunsa 'Tiibii' Akkaataa qajeelfama agarsiisa biyyooleessaatiin fudhachaan jirajettanii yaadduu?

Gaffilee kaka'umsaa

- Iddoon DOTS kun filannoo keessaan jalqabaa yeroo isin gargarsa barbaaddaniitii? Yoo ta'e maaliif? Yoo hin taane, eessa?
- Kunuunsa fayyaa kennitoota waliin iddoo yaalinsa filannoo keessan mariyattanittuu? Yoo lakki, maaliif hin taane?
- Kunuunsa fayyaa kennitootni/gargaartoonni yaalinsaa guyyaa guyyaan yeroo qoricha liqimsanu isin ilaaluu? yoo miti ta`e? maaliif hin taanee ?
- Namoota fayyaa irraatti hojjatan waliin guyya guyyaa walitta dhufeenya keessan natti himuu dandeessu? (walitti haasa'uu,hiriyyummaan?gaaffii gaafachuufi carraa argachuu ? hordoffii , yaalinsa sirrii hin taane)
- Erga yaalinsa eegaltanii; waa'ee 'Tiibii', sababoota 'Tiibii', yaalinsa fi ittisa 'Tiibii'caalaatti waan barattan jiraa? Maddi odeeffannoo keessan eenyu /maalii?
- Kunuunsa Tiibii fudhachuu irraatti rakkoowwan gurguddoo hanga yoonaa isin qunname maal fa'I dha ?

Dhimmaa 3ffaa Yaalinsa waliirra hin cinnee fi bu`aa

1. Mallattoon dhukkubaa TB inni jalqabaa maali jettanii yaaddu?
2. Yaalinsi 'Tiibii' asitti sinii kenname sirrii yookin gahaadha jettanii yaadduu?
3. Sadarkaa kamiirraatti 'Tiibii' irraa fayyeera jettanii kan yaaddanu?

Gaaffilee kaka`umsaa

- Waa'ee yaalisa 'Tiibii' guyyaa guyyaa maal jechuu dandeessu? Yaalinsa kee giddutti kuttee beektaa? Yoo eeyyee ta'e maaliif? yeroo hagamiif?
- Yaalinsa 'Tiibii' addaan kutuun maal fida jettanii yaaddu ?
- Yaalinsa Tiibii ; bu'aa yaalinsa dadhabaa irraatti rakkoolee fi haala mijataa ta'an nuufi ibsuu dandeessuu?

9. Yaalinsi 'Tiibii' kilinika fayyaa keessatti kennamaa jiruu fi hawaasa keessatti kennamaa jiru jidduu garagarummaan jiru maal jettanii yaaddu? (haal qindeessichaa qorataan fakkeenya kennuu qaba.)
Akkaataa yaaliinsa itti qindeessan irratti gargaarumaa fi walfakkeenyi barbaachisaa jira jettanii yaadduu?

10. Yaalinsa Tiibii amma jiru foyyeessufi yaadota akkamii kennitu?

11. Kunuunsa Tiibii jiru ilaalchisee yaada ykn odeeffaannoo biroo qabduu ?

4. Agarsiisa Gaaffii fi deebii hojii gageessaa sagantaa /gaggeessa biiroo fayyaa naannoo

Odeeffannoo Dub jalee

Saalaa -----------------umurii --------------------ogummaa --------------------------sanganticha irratti- itti gaafatamummaa ---------------------leenjii 'Tiibii' DOTS-------------------------------yoo eeyyee ta'e , yeroo hangamiif?-------------------------------------

Seensaa/Gaaffii galumsaa

1. Maaloo, sagantaa to'annaa biyyooleessaa 'Tiibii' Muuxannoo keessaan natti himuu dandeessuu?

Dhimma 1. Bu`uuralee misoomaa fi qabeenyaa biroo

2. Naannoo oromiyaa keessaatti 'Tiibii' DOTS raawwachiisuudhaafi waa'ee jiraachuu bu'uuralee misoomaa fi qabeenya biroo barbaachisaa ta'an maal yaadduu?

Gaaffilee kaka'umsaa

- Naannoo oromiyaa jalatti godinaalee jiran hundumaafi haala tamsaasaa akkamitti ilaaltu?
- Waa'ee madda galii/qabeenyotaa natti himuu dandeessuu?
- Sagantichaafi barbaachisaa ta'ee osoo jiruu misooma bu'uuraa /qabeenyaa dhabame naaf ibsuu dandeessuu?
- Bu`uuralee misoomaa fi qabeenyaa biroon walqabate haala mijeessitootni kam faadha?
- Bu`uraalee misoomaa fi qabeenya biroon walqabtan haalli rakkisoon kam faadha?
- Qabeenyawwan godinaalee adda addaatti DOTS iif ramadaman itti fayyadama isaa akkamitti ilaaltu? Keessattuu kan godina jimmaa ?

Dhimma 2. Akkaataa agarsiisa biyyooleessaan hojjachuu

1. Naannoo oromiyaatti raawwatiinsa waliigalaa DOTS akkamitti ilaaltu?Kan godina jimmaa akkamitti ilaaltu?

Gaffilee kaka'umsaa

- Namoonni fayyaa irratti hojjatan akkaatadhuma agarsiisa biyooleessaatiin hojjataa jiru jettani yaadduu? (yerootti,guutiinsaa,sirrummaan gabaasuu, kkf)
- Agarsiisa biyyooleessaatiin hojii irraa oolmaa kunuunsa fayyaa kennitootaaf rakkooleen jiran maal faadha?
- Agarsiisabiyyooleessaatiin hojiirra oolmaaa kunuunsa fayyaa kennitootaaf haala mijeessitoota kamfaa dhaa ?

- Bara kana supparviizyinii gargaarsaa rawwattee beektaa? yeroo hagamiif? Dub-deebiin bareeffamaan/afaaniin jira turee?

Dhimmaa 3ffaa Bu`aa DOTS

4. Naannoo oromiyaatti waa'ee raawwatiinsa waliigalaafi Bu'aa DOTS maal yaaddu? Godina jimmaattoo?

5. Godinaa/aanaa kana keessaatti bu'aa yaalinsa 'Tiibii' ilaalchisee muuxannoo fi yaada keessan maaloo natti himuu dandeessuu?

Gaaffilee kaka`umsaa

- Raawwatiinsa waliigalaa akkamitti ilaaltu?
- Bu'aa DOTS akkamitti ilaaltu? Akka eegamuu?
- Bu'aa kanaafi sababootni/ibsamni maalfaadha
- Bu'aa mijaawaata'eefi rakkoowwaniifi haala mijeessi ta'uudanda`a maali dha jettanii yaaddu?

6. Naannoo oromiyaatti 'DOTS'iin haawaasa bu`uureeffatee fi dhaabbata bu`ureeffate jidduu garaagarummaan isaanii maal yaaddu? Lamaanuu hojiiatamaa jiruu?

7. Muxannoo keessaan keessatti garaagarummaa ilaalachiseefakkeenyaaf Qulqullina yookiin milkaa'inaa jidduujaraa jiraayi? Eeyyee yoo ta'e, garaagarummaan kun maaliif akka jiraate ibsuu dandeessu?

8. Sagantaa To'annaa 'Tiibii' amma jiru fayyeessuuf yaadota maali kennitu?

9. Tarsiimoo 'DOTS' ilaachisee yaadawwan ykn odeeffaannoo biroo kamiyyuu kennuu barbaaddan qaduuyii?
