# Supplementary material for: Barriers for tuberculosis case finding in Southwest Ethiopia: A qualitative study
Source: PLoS One. 2020 Jan 2;15(1):e0226307. doi: 10.1371/journal.pone.0226307 (PMC6939902; doi:10.1371/journal.pone.0226307)
Supplement: S2 Text — (PDF) [file pone.0226307.s002.pdf]

A Checklist for availability of infrastructure and other resources

Instruction: This checklist should be completed by observing the health facilities and asking persons in charge. The presence and absence should be verified through observation.

Permission for observation and interview

Dear Sir/Madam

My name is **---------------------------------------.** I am a member of a study team conducting research on performance and quality of Tuberculosis Directly Observed Treatment Short course (TB DOTS) strategy in Jimma Zone. The aim of this study is to assess the over performance and quality of TB DOTS strategy. Then to provide important information which will help for program improvement. The knowledge provided from this study will help us to provide useful information for decision makers so that the program performance and quality can be improved and the community members may get better services. Your District is randomly selected for this study. I will ask you to provide some information about the program resources. The information you will provide for us will be handled straightly in a confidential manner and will not be shared with anyone with your identity or identify you as the source. I would like to ask you your permission so that I can observe the infrastructure and other resources related to TB control program. Also the information collected through this observation will be kept strictly confidential.

The project is approved by the Regional Ethical Committee, South-East, in Norway, and the Institutional Review Board Ethical in Jimma University, Ethiopia.

Can I get your cooperation in providing necessary information and documents?

1. Yes ---------- 2. No -------------

Name and signature of data collector _______________________________ Date ________________

Name and signature of data supervisor ___________________________ Date ________________

DOTS site/ Health office --

**Over all infrastructure and patient environment**	Yes	No	NA*	Remark
Does the facility have full time DOTS provider?				
Does the facility have trained laboratory personnel? (on AFB techniques)				
Is there waiting area for patient?				
Does the facility have separate and equipped room for TB clinic?				
Does the health facility have safe water supply?				
Does the health facility have electricity?				
Is appropriate sign posted to locate TB clinic?				

**Over all infrastructure and patient environment …**	Yes	No	NA*	Remark
Are there information education and Communication (IEC) materials posted at patient waiting area and OPD room at visible place in local language				
**Outpatient departments (OPDs)for availability of the following**				
National TB control program guideline				
Laboratory AFB request paper				
Drug prescription paper				
Usable OPD abstract				
**Laboratory unit for availability of the following**				
National TB control program guideline				
Standard operating procedures of AFB (SOPs)				
IEC material posted on visible place in local Language?				
Laboratory AFB register				
Carbon fuchsine				
Acid alcohol				
Methylene blue				
Functional Microscope (Write type of microscope as remark)				
Sink with running water				
Alarm clock				
Staining rack				
Drying rack				
Spirit lamp				
Forceps				
Slides (Write type of slides as remark)				
Sputum cups				
Immersion oil				
Lens tissue				
Disinfectant (either 5% phenol or 10% sodium hypo chloride)				
Filter paper				
Applicator stick /wire loop				
Glove				
**TB clinic** (check for expiry date for the drugs)				
Is TB consultation room clean?				
Is there adequate light in TB room?				

**TB clinic …**	Yes	No	NA*	Remark
Is there adequate ventilation in TB room?				
Are there IEC materials with local language posted at visible spaces?				
Is there NTCP manual?				
Is there reporting formats				
Is there functional standard unit register?				
Is there functional weighting scale?				
Is there RHZE(Rifampicin,Isoniazid, Pyrazinamid, Ethambutol)				
RHZ (Rifampicin,Isoniazid, Pyrazinamid				
Is there RH (Rifampicin,Isoniazid)				
Is there Streptomycin (S)				
Does TB room provide privacy?				
Is there Masks				
Is there appropriate safety box				
**Pharmacy Store for availability of the following** (check for expiry date for the reagents and drugs)				
RHZE				
RHZ				
RH				
Streptomycin (S)				
Sputum cup				
Slides				
Carbol fuchsine				
Methylene blue				
Acid alcohol				
Immersion oil				
Applicator stick				
**Review stock card for the following if stock out for the last three months**				
RHZE				
RHZ				
RH				
Streptomycin (S)				
Sputum cup				

**Review stock card …**	Yes	No	NA*	Remark
Slides				
Carbol fuchsine				
Methylene blue				
Immersion oil				
Applicator stick				
**Check availability of stock for at least the next two months**				
RHZE				
RH				
Streptomycin (S)				
Sputum cup				
Slides				
Carbol fuchsine				
Methylene blue				
Acid alcohol				
Immersion oil				
Applicator stick				

NA* - Not applicable

Description of Health facility /DOTS site and rooms

____-----------------------

-- ----------------

Other interesting observations (environment)

____

____

____

____
