# Supplementary material for: Barriers for tuberculosis case finding in Southwest Ethiopia: A qualitative study
Source: PLoS One. 2020 Jan 2;15(1):e0226307. doi: 10.1371/journal.pone.0226307 (PMC6939902; doi:10.1371/journal.pone.0226307)
Supplement: S3 Text — (PDF) [file pone.0226307.s003.pdf]

COREQ (COnsolidated criteria for REporting Qualitative research) Checklist

Topic	Item No.	Guide Questions/Description	Reported on
**Domain 1: Research team and reflexivity**			
Personal characteristics			
Interviewer/facilitator	1	Which author/s conducted the interview or focus group?	Page 7, materials and method section, study participant and data collection sub section
Credentials	2	What were the researcher's credentials? E.g. PhD, MD	NA
Occupation	3	What was their occupation at the time of the study?	NA
Gender	4	Was the researcher male or female?	Page 9, materials and method section, data analysis, data quality and trustworthiness sub section
Experience and training	5	What experience or training did the researcher have?	NA
Relationship with participants			
Relationship established	6	Was a relationship established prior to study commencement?	Page 7, materials and method section, study participant and data collection sub section
Participant knowledge of the interviewer	7	What did the participants know about the researcher? e.g. personal, goals, reasons for doing the research	Page 9, materials and method section, ethical considerations sub section
Interviewer characteristics	8	What characteristics were reported about the inter viewer/facilitator? e.g. Bias, assumptions, reasons and interests in the research topic	Page 5 and 9, introduction section, and materials and method section, data analysis, data quality and trustworthiness sub section
**Domain 2: Study design**			
Theoretical framework			

Topic	Item No.	Guide Questions/Description	Reported on
Methodological orientation and Theory	9	What methodological orientation was stated to underpin the study? e.g. grounded theory, discourse analysis, ethnography, phenomenology, content analysis	Page 8, materials and method section, data analysis, data quality and trustworthiness sub section
Participant selection			
Sampling	10	How were participants selected? e.g. purposive, convenience, consecutive, snowball	Page 6, materials and method section, sample size and sampling techniques sub section
Method of approach	11	How were participants approached? e.g. face-to-face, telephone, mail, email	Page 7, materials and method section, study participant data collection subsection
Sample size	12	How many participants were in the study?	Page 6, materials and method section, sample size and sampling techniques sub section
Non-participation	13	How many people refused to participate or dropped out? Reasons?	N/A
Setting			
Setting of data collection	14	Where was the data collected? e.g. home, clinic, workplace	Page 7, materials and method section, study participant data collection subsection
Presence of non-participants	15	Was anyone else present besides the participants and researchers?	Page 9, materials and method section; data analysis, data quality and trustworthiness sub section
Description of sample	16	What are the important characteristics of the sample? e.g. demographic data, date	Page 10, result section, table 1
Data collection			
Interview guide	17	Were questions, prompts, guides provided by the authors? Was it pilot tested?	Page 7, study participant and data collection subsection and page 9, data analysis, data quality and trustworthiness sub section
Repeat interviews	18	Were repeat inter views carried out? If yes, how many?	N/A
Audio/visual recording	19	Did the research use audio or visual recording to collect the data?	Page 8, materials and method section, study participants and data collection subsection

Topic	Item No.	Guide Questions/Description	Reported on
Field notes	20	Were field notes made during and/or after the interview or focus group?	Page 8, materials and method section, study participants and data collection subsection
Duration	21	What was the duration of the inter views or focus group?	Page 6, materials and method section, study setting and period subsection
Data saturation	22	Was data saturation discussed?	Page 9, materials and method section, data analysis, data quality and trustworthiness subsection
Transcripts returned	23	Were transcripts returned to participants for comment and/or correction?	No, but peer debriefing done by listing the de-identified audio taped interviews and checking with the transcripts, Page 9, materials and method section; data analysis, data quality and trustworthiness subsection
**Domain 3: analysis and findings**			
Data analysis			
Number of data coders	24	How many data coders coded the data?	Page 8, materials and method section; data analysis, data quality and trustworthiness subsection
Description of the coding tree	25	Did authors provide a description of the coding tree?	No
Derivation of themes	26	Were themes identified in advance or derived from the data?	Page 8, materials and method section; data analysis, data quality and trustworthiness subsection
Software	27	What software, if applicable, was used to manage the data?	Page 8, materials and method section; data analysis, data quality and trustworthiness subsection
Participant checking	28	Did participants provide feedback on the findings?	N/A

Topic	Item No.	Guide Questions/Description	Reported on
Reporting			
Quotations presented	29	Were participant quotations presented to illustrate the themes/findings?<br>Was each quotation identified? e.g. participant number	Page 11- 16, result section
Data and findings consistent	30	Was there consistency between the data presented and the findings?	Page 12 and 13, result section
Clarity of major themes	31	Were major themes clearly presented in the findings?	Page 11, result section
Clarity of minor themes	32	Is there a description of diverse cases or discussion of minor themes?	Page 11 and 14, result section, inadequate resources for TB case finding subsection and limited access to TB diagnostic service subsection
