# Supplementary material for: Barriers for tuberculosis case finding in Southwest Ethiopia: A qualitative study
Source: PLoS One. 2020 Jan 2;15(1):e0226307. doi: 10.1371/journal.pone.0226307 (PMC6939902; doi:10.1371/journal.pone.0226307)
Supplement: Attachment — Submitted filename: Response to Reviewers .pdf [file pone.0226307.s006.pdf]

PONE-D-19-23348

Barriers for tuberculosis case finding in Southwest Ethiopia: A qualitative study

PLOS ONE

First of all, we would like to thank the Academic Editor for his careful reading, and the Reviewers for their high quality and constructive reviews of our manuscript. Sincerest thank you so much for your prompt response on our manuscript.

We have modified the manuscript in response to the extensive and insightful reviewers' comments. We hope that a revised version of the manuscript will still be considered by PLOSONE. A detailed point-by-point response to each of the Reviewers' comment are as follow:

Reviewer #1 (R1):

Summary

R1- The manuscript looks at a great deal of data collected from 60 in-depth interviews with health workers, program managers, and patients as well as clinic observations from 42 facilities to determine reasons for a low TB notification rate in a rural area of Ethiopia. The researchers have done a great deal of work to analyse and triangulate interview and observational data, showing where clinic resources fall short. On the whole, this is a well-researched manuscript and will provide a good addition to the qualitative literature on TB in low-resource settings once revisions are made to reduce repetition throughout the manuscript and strengthen the interpretation of findings.

Response – Thank you so much for your constructive comments. We have critically reviewed the manuscript and reduced the repetitions.

Suggestions for major revision:

R1- The manuscript could use a critical trim, as each of the sections have a lot of repetition. For example, the description of facility observation is described in multiple paragraphs of methods. In several places throughout the manuscript, two sentences will be used to say the same thing.

Response – Thank you so much for your insightful comment. We are sorry for this mistake. We have reduced the repetitions by reorganizing the paragraphs and deleting unnecessary repetitions.

R1- A second issue relates to one of the themes identified in the findings, namely 3.3. I am unconvinced by the data that there is a lack of information on TB in the community. Health workers often blame health delays on patients, whereas patients often recount long journeys to diagnosis once they make contact with the system. Often there is delay at both patient and facility level; however, the data presented on patient delay in your study is conjecture. Only 10 patients were interviewed – an unlikely number to reach saturation – and those interviewed did not report long delays between symptom onset and visiting a health facility. Both health workers and patients alike thought that more could probably be done to improve knowledge in the community, but the data reported does not support this finding. As such, I would advise removing the discussion on patient education and removing comparisons to other studies in which patient delay was noted – this is unrelated to your study, and you acknowledge the lack of data from patients not reaching clinic as one of your study limitations.

Response – We appreciate your insightful suggestions. We agree that 3.3 (a lack of information on TB in the community) theme was only perceived barrier from health workers' and patients' view and not assessed from the community. We have modified the manuscript in line with the reviewer's comments.

R1- Furthermore, I would like to push the authors a bit further in terms of discussion and recommendations. To add substantively to the qualitative literature, it is insufficient to recommend more resources and more education when resources are limited and there is a plethora of research to show that more education does not necessarily lead to changes in practice. Your interview findings on resource shortages are important and are well backed by the clinic observation. The next step is to place these findings in context for the reader, e.g. what are the particular circumstances and potential ways to improve the situation within the existing context and resources.

Response - Thank you so much. As you suggested, we have modified the discussion and included more feasible (in terms of resources and practicability) recommendations with available resources and for Ethiopian context.

R1- Does the existing data include health worker perspectives on why there is such high staff turnover (not just conjecture from program managers)? Money and risk aversion are not the only

motivators. It would be interesting to see some perspectives from the DOT workers on what motivates them to stay or prompts them to move on, if such data is available.

Response - Thank you so much for your constructive comment. It is the perspectives of both program managers and DOT providers as you see from the result section of 3.1.1. Moreover, the issue of incentives (not only money but also moral like chance of education) was mentioned by the DOT providers.

R1- Furthermore, while the data is compelling to show that there is a lack of quality decentralised services in the region of study, it would be useful to discuss models of decentralisation in low-resource settings that could be implemented in the Ethiopian context. It also may not be reasonable to expect a lab technician to be available to conduct AFBs in all clinics, but building a better supply chain despite poor roads and long distances has been successful in other settings.

Response – We appreciate your insightful suggestions. We have included under implications for practice (recommendations) section as per your comments.

R1- Rebuilding existing centres may not be economically viable, but building future clinics with a mind to separating potentially active TB patients from other sick patients may be an important recommendation, as well as identifying other low-cost strategies that have been used elsewhere to ensure safety of other patients in waiting rooms and during sputum collection – for example having patients go outside to a private area to bring up sputum and having chairs available outside the clinic or providing basic masks for any coughing patients while in existing waiting areas to reduce nosocomial transmission (although this also requires a degree of sensitisation to prevent stigma). Try to find examples of these in the literature to provide practical recommendations, or tie your findings to existing theories in new ways that will help policymakers and providers think of new ways to approach the issues identified.

Response - We appreciate your insightful suggestions. We have modified the recommendations by including low cost strategies like construction of patient waiting area and sputum collection area by mobilizing the community to avail or contribute locally available resources such as wood, stone, as well as free labor.

Minor suggestions and editorial corrections:

R1- Introduction:

It would be helpful to have an overview of correct TB management in the Ethiopian context. In South Africa, for instance, sputum is first sent for GeneXpert when PTB is suspected, whereas reference is made to stock outs of AFB. It would help the reader to understand the basic TB testing algorithm in use during your study.

Response - Thank you so much. We have included an overview of correct TB management in the Ethiopia in introduction.

R1- The use of DOT and DOTS are at times confused throughout the manuscript. DOT refers to the act of directly observing TB treatment, whereas DOTS refers to the programme, which involves not just the observation of treatment, but supply chain management, testing facilities, and political will, etc. Be careful not to mix these up when referring to frontline DOT workers versus those who make up other aspects of the DOTS strategy.

Response – Thank you so much for noting this. The use of these terms has been edited and corrected throughout the manuscript.

R1- Be cautious of the language used to describe patients. The use of terms like "TB suspects" and "defaulting" imply wrong-doing or blame, when TB transmission and loss to follow-up are more systemic issues. For example Line 74: "TB suspect cases" should be changed to "People who screen positive for TB" and in Line 80: "Treatment default rate" is more appropriately referred to as loss to follow-up of those on treatment.

Response – Thank you so much for noting these. These terms has been edited and replaced with proper terms in line with your comments.

R1- Line 62-63: quality of TB care at the primary care level is another (needs a period)

Response – We have included a period.

R1- Line 93: they also detecting close contact of (incorrect grammar) / please define close contacts.

Response – the term was clarified.

R1- Line 95: remove the ‘s’ from passive case findings

Response –‘s’ was removed.

R1- Line 95-98: This sentence is long and confusing. Consider revising.

Response - The sentence has been revised.

R1- Line 106-107 repeat definition, plus the bracketed information is incorrect - it is time from first symptoms to diagnosis, as stated previously. Please delete

Response – The repeated and incorrect information has been deleted.

R1- How were notification and case rates determined?

Response:

For TB case notification rate:

Numerator: Number of new and relapse TB cases reported in the past year (×100,000)

Denominator: Total population in the specified area

For TB case detection rate:

Numerator: Number of new TB cases detected

Denominator: Estimated number of new TB cases countrywide

R1- Line 109: unpublished annual report – annual reports should be referenced appropriately

Response: The annual report has been cited and referenced accordingly.

Methods:

R1- Line 121-22, the number of districts and administrative towns should not be referenced as an observation.

Response – It has been corrected

R1- Line 124: “In 2016 it has” change ‘has’ to ‘had.’

Response – It has been changed.

R1- Line 127 The phrase “TB suspect identification” should read “TB screening”

Response – The phrase has been corrected accordingly.

R1- Line 129, what is the definition of coverage being used?

Response: It was measured by ratio of health facilities to the population

R1- Line 133: Since the CDC is a government organisation, I would suggest replacing NGO with global health agencies.

Response – It was replaced as per your comment.

R1- Line 137: The correct terminology is purposive selection (not purposely selected).

Response - The terms have been corrected accordingly.

R1- Line 164 “Secondly, a facility observations” should read “Secondly, facility observations” (remove ‘a’)

Response - ‘a’ has been removed and the sentence has been taken to proper paragraph.

R1- As Table 1 describes demographics, it would normally be placed under the results section.

Response- Table 1 has been moved to the result section.

R1 - Section 2.4 uses quotations around qualitative research concepts such as “thick description” and “optimal” distance as insider-outsider to the data. These concepts should be referenced from the literature.

Response – We have included the references for the concepts.

Results:

R1- Line 248-250, it would read more clearly if the authors stated that “three main themes were identified from the data: 1, 2, 3,” rather than listing the themes in brackets. Although a personal preference, I also try to avoid the use of phrase “themes emerging from the data,” as this seemingly undermines the difficult task of the researchers whose work it is to identify the themes after a lengthy analysis process.

Response – Thank you so much for your wonderful view. We have modified as per your comments.

R1- Line 340: add an ‘s’ to causes: “They also mentioned other cause”

Response - ‘s’ has been added.

R1- Line 350: replace “told” with “spoke about”

Response- The term has been replaced.

R1- Line 351: replace “was” with “were”

Response- The term has been replaced.

R1- Lines 375-76 This sentence is confusing in the use of “few”. Perhaps it would be clearer to say “to explain the paucity of decentralised sites providing diagnostic services”

Response – The sentence has been modified as per your suggestion.

R1- Section 3.2.1: There is a difference between having access, i.e. existence of a nearby health facility and gaining access, i.e. accessibility as in feeling safe and supported at health facilities. You refer to this in the discussion. Did this difference come up in any of the patient interviews?

Response - It was limited **access** to diagnostic services since access include availability, accessibility, and accommodation dimensions which were reflected in our finding.

Discussion:

R1- In response to 3.2.1: Recently there has been a larger focus placed on the quality of TB care once reaching the facility (versus access), i.e. health workers thinking to test for TB as well as tests being affordable and available. The continued use of AFB, which has very poor sensitivity compared to GeneXpert is also of concern. It is important to touch on these aspects in the discussion.

Response – We have included these issues in the discussion section

R1- Lines 463-465: Referring to what works in Sweden, a high-income, low-burden country is not necessarily transferable to the Ethiopian context. Are there other more relevant references in this regard?

Response - Thank you so much for your valuable comment. We have incorporated similar studies and context in the discussion section.

R1- Line 529: First use of the acronym HEWs. Use consistently throughout or not at all.

Response - It was corrected accordingly.

Reviewer #2 (R2):

General comments

In the abstract section

R2- 1. Results: "Inadequate resources for TB case finding"; this is a general term and the authors should describe the resources that are inadequate for TB case finding briefly.

Response - Thank you so much for your constructive comments. We have included a brief description

R2- 2. ......inaccessibility to TB diagnostic facilities...... this should also be clearly described because accessibility/access has various dimensions. Is this physical inaccessibility, affordability, acceptability or availability?

Response - Thank you so much for your insightful comments. We have included a description and it was limited **access** to diagnostic services since access include availability, accessibility, and accommodation dimensions which were reflected in our finding.

R2. 3. Conclusion:..... "Limited resources for TB case finding"..... 'Inadequate resources for TB case finding' in the results section.

Response- Thank you so much for your useful comment. We have made consistent both at result and conclusion.

Introduction

R2- 4. The authors should check the use of full stop they used it before the references. It was not consistent. For instance, on line number 44 and on others, the full stop was used before references.

Response - Thank you so much constructive comment. As you suggested, we have moved the full stop after the references throughout the manuscript.

R2- 5. The authors used 2015's data or report; it would have been better to use the recent reports of TB case notification rates of Ethiopia for global comparisons.

Response – Thank you so much for the comment. We would like to clarify this. We have used 2016 data or report since the study was conducted from August 2016 to January 2017. Which means we had to identify the gap or problem before conducting the study.

R2- 6. Line number 123, why the authors could not put the number of health posts? They reported approximately 500 despite knowing the number of all kebels.

Response – The exact number of the health posts during the study period has be included.

R2- 7. The health service coverage of the Zone reported to be approximately 52%. Is this based on physical coverage or health facility density to population ratio? The total population of the Zone was 2,607,115, the number of health facilities was also known; the authors should check the population to health centre density or other facilities as well; 2, 607115/123. One health centre is expected to serve 25000 people. Moreover, DOTs and TB diagnostic services coverage should also be described.

Response - We appreciate your insightful suggestions. It was health facility density to population ratio. We have included TB diagnostic services coverage and health posts' DOT coverage.

Methods

R2- 8. Table 1 under "Duration of experience for current Responsibility" the authors mentioned only <2years, 2-5 years and 9-10 years; why the authors didn't mention those who had 6-8 years and above ten years of experience?

Response – Thank you so much for your careful review. We are sorry for this mistake. It was typographical error and has been corrected.

R2- 9. Line 189, " A relaxed atmosphere".... this is not clear for a new reader and I would suggest using another term.

Response – The terms have been clarified.

R2- 10. Line number 189 "frequent discussions and consultations among the authors"..........did not specify why they did the discussion and consultations; the discussions were about what....?

Response - Thank you so much for your comment. Reasons for discussions and topic of discussions have been included.

R2- 11. Line numbers 231 and 234-235; are the research assistant and interviewer different or are the same people? This is not clear.

Response - The interviewer was first author (BME) and the research assistant was who assisted the data collection of facility observation. We have clarified in the manuscript.

Results

R2- 12. In line numbers 265-269 the authors mentioned different reasons for shortage of laboratory technicians. One of this was the "due to a closed laboratory program in some training institutions (Ethiopian politicians considering the saturation of laboratory professionals in the country)" .... what about the problems in maldistribution of the available human health workforce. Did the authors see for evidence of urban concentration and rural scarcity? This could also affect the availability and contributes to inequities which in turn affect TB control performance. In some rural settings even if the professionals are available in the job market budget constraints to hire the professionals could be one of the challenges.

Response – We have included about the maldistribution. However, the program managers mentioned that they couldn't get laboratory professionals easily in the job market even if they had the budget.

R2- 13. Under "inadequate basic infrastructure"..... it would be better if the authors mention the numbers or proportions instead of ...... " The majority of health posts".......... "several health centers".... "most health centers"........

Response – Thank you so much. We have included number or percentage based on observation finding.

R2- 14. Discussion: the strengths and limitation of the study design as well as possible bias and measures taken to reduce the bias were not discussed. The authors should consider this in their discussion.

Response – Thank you so much for your constructive comment. We have included the points as per your comment.
