# Supplementary material for: Barriers for tuberculosis case finding in Southwest Ethiopia: A qualitative study
Source: PLoS One. 2020 Jan 2;15(1):e0226307. doi: 10.1371/journal.pone.0226307 (PMC6939902; doi:10.1371/journal.pone.0226307)
Supplement: Attachment — Submitted filename: Response to Reviewers .pdf [file pone.0226307.s007.pdf]

PONE-D-19-23348

Barriers for tuberculosis case finding in Southwest Ethiopia: A qualitative study

PLOS ONE

First of all, we would like to thank the Academic Editor for his careful reading, and the Reviewers for their high quality and constructive reviews of our manuscript. Many thanks for your prompt response.

We have modified the manuscript in response to the extensive and insightful reviewers' comments. We hope that a revised version of the manuscript will still be considered by PLOSONE. Detailed point-by-point responses to each of the Reviewers' comment are as follow:

Reviewer #1 (R1):

Summary

R1- The manuscript looks at a great deal of data collected from 60 in-depth interviews with health workers, program managers, and patients as well as clinic observations from 42 facilities to determine reasons for a low TB notification rate in a rural area of Ethiopia. The researchers have done a great deal of work to analyse and triangulate interview and observational data, showing where clinic resources fall short. On the whole, this is a well-researched manuscript and will provide a good addition to the qualitative literature on TB in low-resource settings once revisions are made to reduce repetition throughout the manuscript and strengthen the interpretation of findings.

Response – Thank you so much for your constructive comments. We have critically reviewed the manuscript and reduced the repetition throughout the manuscript particularly the "Method", and "Result" sections, pages 6 – 16.

Suggestions for major revision:

R1- The manuscript could use a critical trim, as each of the sections has a lot of repetition. For example, the description of facility observation is described in multiple paragraphs of methods. In several places throughout the manuscript, two sentences will be used to say the same thing.

Response – Thank you so much for your insightful comment. We are sorry for this mistake. We have corrected by reorganizing the paragraphs and deleting unnecessary

repetitions. This is located at “Method”, section, “study participants and data collection” sub-section, page 7 and 8.

R1- A second issue relates to one of the themes identified in the findings, namely 3.3. I am unconvinced by the data that there is a lack of information on TB in the community. Health workers often blame health delays on patients, whereas patients often recount long journeys to diagnosis once they make contact with the system. Often there is delay at both patient and facility level; however, the data presented on patient delay in your study is conjecture. Only 10 patients were interviewed – an unlikely number to reach saturation – and those interviewed did not report long delays between symptom onset and visiting a health facility. Both health workers and patients alike thought that more could probably be done to improve knowledge in the community, but the data reported does not support this finding. As such, I would advise removing the discussion on patient education and removing comparisons to other studies in which patient delay was noted – this is unrelated to your study, and you acknowledge the lack of data from patients not reaching clinic as one of your study limitations.

Response – We appreciate your insightful suggestions. We agree that 3.3 (a lack of information about TB in the community) theme was only perceived barrier from health workers’ and patients’ view and not assessed from the community. We have modified the manuscript in line with the reviewer’s comments by removing it from the “Result”, “Discussion”, and “Implications for practice” sections.

R1**-** Furthermore, I would like to push the authors a bit further in terms of discussion and recommendations. To add substantively to the qualitative literature, it is insufficient to recommend more resources and more education when resources are limited and there is a plethora of research to show that more education does not necessarily lead to changes in practice. Your interview findings on resource shortages are important and are well backed by the clinic observation. The next step is to place these findings in context for the reader, e.g. what are the particular circumstances and potential ways to improve the situation within the existing context and resources.

Response - Thank you so much. As you suggested, we have modified the discussion and included more feasible recommendations with available resources and for Ethiopian context. This is located at “Discussion” section, last paragraph of page 19

lines 512 - 516; and “Implications for practice” section, 1st paragraph page 21, lines 549 and 550, 2nd and page 22 lines 580 – 583, 590 and 591.

R1- Does the existing data include health worker perspectives on why there is such high staff turnover (not just conjecture from program managers)? Money and risk aversion are not the only motivators. It would be interesting to see some perspectives from the DOT workers on what motivates them to stay or prompts them to move on, if such data is available.

Response - Thank you so much for your constructive comment. The issue of “staff turnover” was the perspectives of both program managers and DOT providers. Moreover, the issue of incentives (not only money but also moral like education opportunity) was mentioned by the DOT providers. This is located at “Result” section, “Shortage of health-care providers” subsection, last paragraph of page 11, lines 278 - 281.

R1- Furthermore, while the data is compelling to show that there is a lack of quality decentralised services in the region of study, it would be useful to discuss models of decentralisation in low-resource settings that could be implemented in the Ethiopian context. It also may not be reasonable to expect a lab technician to be available to conduct AFBs in all clinics, but building a better supply chain despite poor roads and long distances has been successful in other settings.

Response – We appreciate your insightful suggestion. We have included best practice on process of decentralization of TB care from low resource setting, and more practical recommendations. This is located under “implications for practice” section, 1st and 2nd paragraphs of page 21, lines 549 and 550, 552 – 560.

R1- Rebuilding existing centers may not be economically viable, but building future clinics with a mind to separating potentially active TB patients from other sick patients may be an important recommendation, as well as identifying other low-cost strategies that have been used elsewhere to ensure safety of other patients in waiting rooms and during sputum collection – for example having patients go outside to a private area to bring up sputum and having chairs available outside the clinic or providing basic masks for any coughing patients while in existing waiting areas to reduce nosocomial transmission (although this also requires a degree of sensitisation to prevent stigma). Try to find examples of these in the literature to provide practical

recommendations, or tie your findings to existing theories in new ways that will help policymakers and providers think of new ways to approach the issues identified.

Response - We appreciate your useful suggestions. We have modified the recommendations by including low cost strategies like construction of patient waiting area and sputum collection area by mobilizing the community to avail or contribute locally available resources such as wood, stone, as well as free labor. This is located under "implications for practice" section, 3rd paragraph page 21, lines 561 and 562, and 2nd paragraph of page 22, lines 580 - 583.

Minor suggestions and editorial corrections:

Introduction:

R1- It would be helpful to have an overview of correct TB management in the Ethiopian context. In South Africa, for instance, sputum is first sent for GeneXpert when PTB is suspected, whereas reference is made to stock outs of AFB. It would help the reader to understand the basic TB testing algorithm in use during your study.

Response - Thank you so much. We have included an overview of correct TB management in the Ethiopia at "Introduction" section, 3rd paragraph of page 4 lines 92 - 101.

R1- The use of DOT and DOTS are at times confused throughout the manuscript. DOT refers to the act of directly observing TB treatment, whereas DOTS refers to the programme, which involves not just the observation of treatment, but supply chain management, testing facilities, and political will, etc. Be careful not to mix these up when referring to frontline DOT workers versus those who make up other aspects of the DOTS strategy.

Response – Thank you so much for noting this. The use of these terms have been edited and corrected throughout the manuscript.

R1- Be cautious of the language used to describe patients. The use of terms like "TB suspects" and "defaulting" imply wrong-doing or blame, when TB transmission and loss to follow-up are more systemic issues. For example Line 74: "TB suspect cases" should be changed to "People who screen positive for TB" and in Line 80: "Treatment default rate" is more appropriately referred to as loss to follow-up of those on treatment.

Response – Thank you so much for noting these. These terms have been edited and replaced with proper terms in line with your comments. This is located at "Introduction" section, 1st and 2nd paragraphs of page 4, lines 80 and 86.

R1- Line 62-63: quality of TB care at the primary care level is another (needs a period)

Response – We have included a period and it is located at "Introduction" section, last paragraph of page 3, line 69.

R1- Line 93: they also detecting close contact of (incorrect grammar) / please define close contacts.

Response – The term was clarified and located at "Introduction" section, 1st paragraph of page 5, line 109.

R1- Line 95: remove the's' from passive case findings

Response –'s' was removed and located at "Introduction" section, 1st paragraph of page 5, line 111.

R1- Line 95-98: This sentence is long and confusing. Consider revising.

Response - The sentence has been revised by making two sentences and deleting unnecessary words. This is located at "Introduction" section, 1st paragraph of page 5, lines 111 - 114.

R1- Line 106-107 repeat definition, plus the bracketed information is incorrect - it is time from first symptoms to diagnosis, as stated previously. Please delete

Response – The repeated and incorrect information has been deleted. This is located at "Introduction" section, 2nd paragraph of page 5, line 122.

R1- How were notification and case rates determined?

Response:

For TB case notification rate: Numerator: Number of new and relapse TB cases reported in the past year (×100,000), and Denominator: Total population in the specified area

For TB case detection rate: Numerator: Number of new TB cases detected and Denominator: Estimated number of new TB cases countrywide.

This is located at "Introduction" section, 2nd paragraph of page 4, lines 84 -85.

R1- Line 109: unpublished annual report – annual reports should be referenced appropriately

Response: The annual report has been cited and referenced accordingly. It is located at "Introduction" section, last paragraph of page 5, line 123.

Methods:

R1- Line 121-22, the number of districts and administrative towns should not be referenced as an observation.

Response – It has been corrected at "Method" section, "Study setting and period" sub-section, and 3rd sentence of page 6, line 137.

R1- Line 124: "In 2016 it has" change 'has' to 'had.'

Response – It has been changed at "Method" section, "Study setting and period" sub-section, page 6, line 139. .

R1- Line 127 The phrase "TB suspect identification" should read "TB screening"

Response – The phrase has been corrected accordingly at "Method" section, "Study setting and period" sub-section, page 6, line142. .

R1- Line 129, what is the definition of coverage being used?

Response: It was measured by ratio of health facilities to the population. This is located at "Method" section, "Study setting and period" sub-section, page 6, line 146. .

R1- Line 133: Since the CDC is a government organisation, I would suggest replacing NGO with global health agencies.

Response – It was replaced as per your comment. This is located at "Method" section, "Study setting and period" sub-section, page 6, line 148. .

R1- Line 137: The correct terminology is purposive selection (not purposely selected).

Response - The term has been corrected accordingly. This is located at "Method" section, first sentence of "Sample size and sampling techniques" sub-section, page 6, line 154. .

R1- Line 164 "Secondly, a facility observations" should read "Secondly, facility observations" (remove 'a')

Response - ‘a’ has been removed and the sentence has been moved to proper paragraph. This is located at “Method” section, last paragraph of “Study participants and data collection” sub-section, page 8,line 192.

R1- As Table 1 describes demographics, it would normally be placed under the results section.

Response- Table 1 has been moved to the result section. This is located at “Result” section, “Characteristics of the study participants” sub-section, page 10.

R1 - Section 2.4 uses quotations around qualitative research concepts such as “thick description” and “optimal” distance as insider-outsider to the data. These concepts should be referenced from the literature.

Response – We have included the references for the concepts. This is located at “Method” section, 3rd and 4th paragraph of “Data Analysis, data quality control and trustworthiness” subsection, page 9, lines 229 and 232.

Results:

R1- Line 248-250, it would read more clearly if the authors stated that “three main themes were identified from the data: 1, 2, 3,” rather than listing the themes in brackets. Although a personal preference, I also try to avoid the use of phrase “themes emerging from the data,” as this seemingly undermines the difficult task of the researchers whose work it is to identify the themes after a lengthy analysis process.

Response – Thank you so much for your wonderful view. We have modified as per your comments. This is located at “Result” section, 1st paragraph of page 11, lines 256 and 257.

R1- Line 340: add an‘s’ to causes: “They also mentioned other cause”

Response - ‘s’ has been added at “Result” section, 2nd paragraph of “Inadequate diagnostic equipment and supplies” sub-section, page 14, line 350.

R1- Line 350: replace “told” with “spoke about”

Response- The term has been replaced at “Result” section, 1st paragraph of “Absence of nearby health facilities providing TB diagnostic service” sub-section, page 14, line 360.

R1- Line 351: replace "was" with "were"

Response- The term has been replaced at "Result" section, 1st paragraph of "Absence of nearby health facilities providing TB diagnostic service" sub-section, page 14, line 361.

R1- Lines 375-76 This sentence is confusing in the use of "few". Perhaps it would be clearer to say "to explain the paucity of decentralised sites providing diagnostic services"

Response – The sentence has been modified as per your suggestion. It is located at "Result" section, last paragraph of "Absence of nearby health facilities providing TB diagnostic service" sub-section, page 15, line 385 and 386.

R1- Section 3.2.1: There is a difference between having access, i.e. existence of a nearby health facility and gaining access, i.e. accessibility as in feeling safe and supported at health facilities. You refer to this in the discussion. Did this difference come up in any of the patient interviews?

Response - It was limited access to diagnostic services since access includes availability, accessibility, and accommodation dimensions which were reflected in our finding. This is located at "Result" section, "Limited access to TB diagnostic service" sub-section, page 14, line 356.

Discussion:

R1- In response to 3.2.1: Recently there has been a larger focus placed on the quality of TB care once reaching the facility (versus access), i.e. health workers thinking to test for TB as well as tests being affordable and available. The continued use of AFB, which has very poor sensitivity compared to GeneXpert is also of concern. It is important to touch on these aspects in the discussion.

Response – We have included studies about these issues. It is located at "Discussion" section, 3rd paragraph of page 19, lines 502 - 511.

R1- Lines 463-465: Referring to what works in Sweden, a high-income, low-burden country is not necessarily transferable to the Ethiopian context. Are there other more relevant references in this regard?

Response - Thank you so much for your valuable comment. We have removed the study in

Sweden, and incorporated similar studies as well as contexts in the discussion section. It is located at “Discussion” section, last paragraph of page 19, lines 512 - 516.

R1- Line 529: First use of the acronym HEWs. Use consistently throughout or not at all.

Response - It was corrected at “Discussion” section, 2nd paragraph of page 19, line 497.

Reviewer #2 (R2):

General comments

In the abstract section

R2- 1. Results: “Inadequate resources for TB case finding”; this is a general term and the authors should describe the resources that are inadequate for TB case finding briefly.

Response - Thank you so much for your constructive comment. We have included a brief description at 3rd paragraph of “Abstract” section, page 2, lines 31 and 32.

R2- 2. ......inaccessibility to TB diagnostic facilities...... this should also be clearly described because accessibility/access has various dimensions. Is this physical inaccessibility, affordability, acceptability or availability?

Response - Thank you so much for your insightful comment. We have included a description and it was limited access to diagnostic services since access includes availability, accessibility, and accommodation dimensions which were reflected in our finding. This is located at 3rd paragraph of “Abstract” section, page 2, lines 33 and 34.

R2. 3. Conclusion:..... “Limited resources for TB case finding”..... ‘Inadequate resources for TB case finding’ in the results section.

Response- Thank you so much for your useful comment. We have made consistent both at result and conclusion. It is located at last paragraph of “Abstract” section, page 2, line 39.

Introduction

R2- 4. The authors should check the use of full stop they used it before the references. It was not

consistent. For instance, on line number 44 and on others, the full stop was used before references.

Response - Thank you so much for your constructive comment. As you suggested, we have moved the full stop after the references throughout the manuscript.

R2- 5. The authors used 2015's data or report; it would have been better to use the recent reports of TB case notification rates of Ethiopia for global comparisons.

Response – Thank you so much for your comment. We would like to clarify this. We have used 2015/6 data or report since the study was conducted in 2016/7. Which means the gap or problem was identified before conducting the study.

R2- 6. Line number 123, why the authors could not put the number of health posts? They reported approximately 500 despite knowing the number of all kebels.

Response – The exact number of the health posts during the study period has been included. This is located at "Method" section, "Study setting and period" sub-section, page 6, line 140.

R2- 7. The health service coverage of the Zone reported to be approximately 52%. Is this based on physical coverage or health facility density to population ratio? The total population of the Zone was 2,607,115, the number of health facilities was also known; the authors should check the population to health centre density or other facilities as well; 2, 607115/123. One health centre is expected to serve 25000 people. Moreover, DOTs and TB diagnostic services coverage should also be described.

Response - We appreciate your insightful suggestion. It was health facility density to population ratio. Also we have included TB diagnostic service coverage and health posts' DOT coverage. This is located at "Method" section, "Study setting and period" sub-section, page 6, lines 143 and 146.

Methods

R2- 8. Table 1 under "Duration of experience for current Responsibility" the authors mentioned only <2years, 2-5 years and 9-10 years; why the authors didn't mention those who had 6-8 years and above ten years of experience?

Response – Thank you so much for your careful review. We are sorry for this mistake. It was typographical error and has been corrected. This is located at "Result" section, table 1, page 10, line 254.

R2- 9. Line 189, "A relaxed atmosphere".... this is not clear for a new reader and I would suggest using another term.

Response – The term has been clarified and located at "Method" section, 2nd paragraph of "Study participant and data collection" sub-section, page 7, line 187.

R2- 10. Line number 189 "frequent discussions and consultations among the authors"..........did not specify why they did the discussion and consultations; the discussions were about what....?

Response - Thank you so much for your comment. Reasons for discussions and topic of discussions have been included. This is located at "Method" section, last paragraph of "Data Analysis, data quality control and trustworthiness" sub-section, page 9, 236 - 238.

R2- 11. Line numbers 231 and 234-235; are the research assistant and interviewer different or are the same people? This is not clear.

Response - The interviewer was first author (BME) and the research assistant was who assisted the data collection of facility observation. We have clarified this at "Method" section, 3rd paragraph of "Data Analysis, data quality control and trustworthiness" sub-section, page 9, line 229.   .

Results

R2- 12. In line numbers 265-269 the authors mentioned different reasons for shortage of laboratory technicians. One of this was the "due to a closed laboratory program in some training institutions (Ethiopian politicians considering the saturation of laboratory professionals in the country)" .... what about the problems in maldistribution of the available human health workforce. Did the authors see for evidence of urban concentration and rural scarcity? This could also affect the availability and contributes to inequities which in turn affect TB control performance. In some rural settings even if the professionals are available in the job market budget constraints to hire the professionals could be one of the challenges.

Response – We have included about the mal-distribution. However, the program managers mentioned that they couldn't get laboratory professionals easily in the job market even if they had the budget. This is located at "Result section", 1st and 2nd paragraphs of "Shortage of health-care providers" subsection, page 11, lines 265, 272 and 273.

R2- 13. Under "inadequate basic infrastructure"..... it would be better if the authors mention the numbers or proportions instead of ...... " The majority of health posts".......... "several health centers".... "most health centers"........

Response – Thank you so much. We have included number or percentage based on observation finding. This is located at Result section, 4th paragraph of "Inadequate basic infrastructure" sub-section, page 13, lines 299 - 302.

R2- 14. Discussion: the strengths and limitation of the study design as well as possible bias and measures taken to reduce the bias were not discussed. The authors should consider this in their discussion.

Response – Thank you so much for your constructive comment. We have included the points as per your comment and located at last paragraph of "Discussion" section, page 20, lines 536 - 545.
